# Supplementary material for: The tumor suppressor microRNA let-7 inhibits human LINE-1 retrotransposition
Source: Nat Commun. 2020 Nov 11;11:5712. doi: 10.1038/s41467-020-19430-4 (PMC7658363; doi:10.1038/s41467-020-19430-4)
Supplement: Supplementary file 1 — Supplementary Information [file 41467_2020_19430_MOESM1_ESM.pdf]

SUPPLEMENTARY INFORMATION

The tumor suppressor microRNA let-7 inhibits human LINE-1 retrotransposition

Tristán-Ramos et al.

Supplementary Figure 1

a

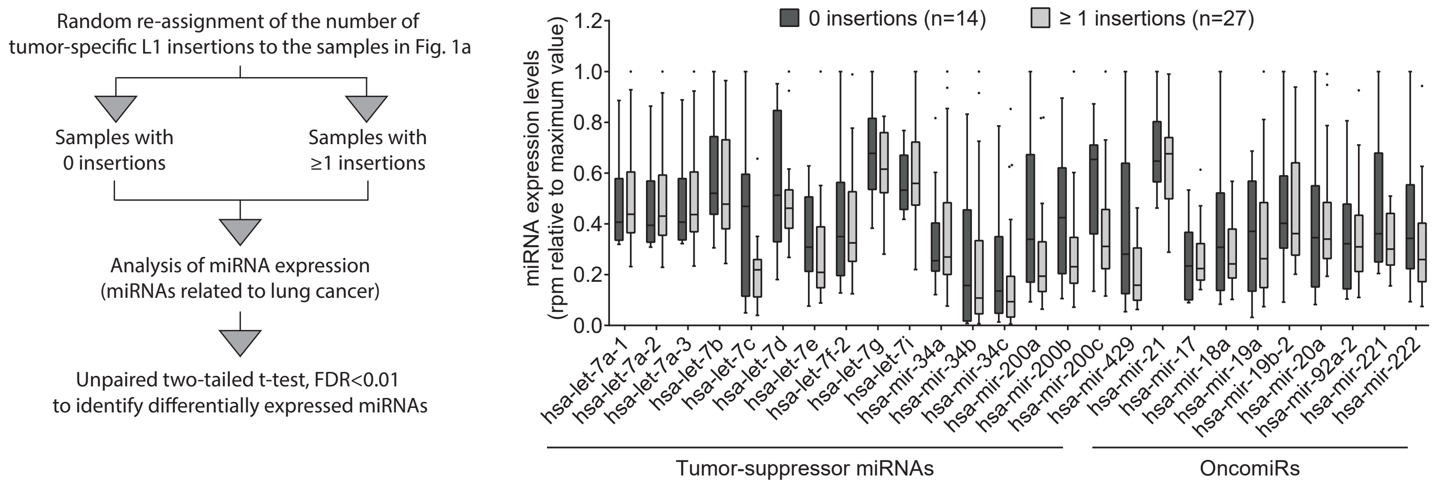

b

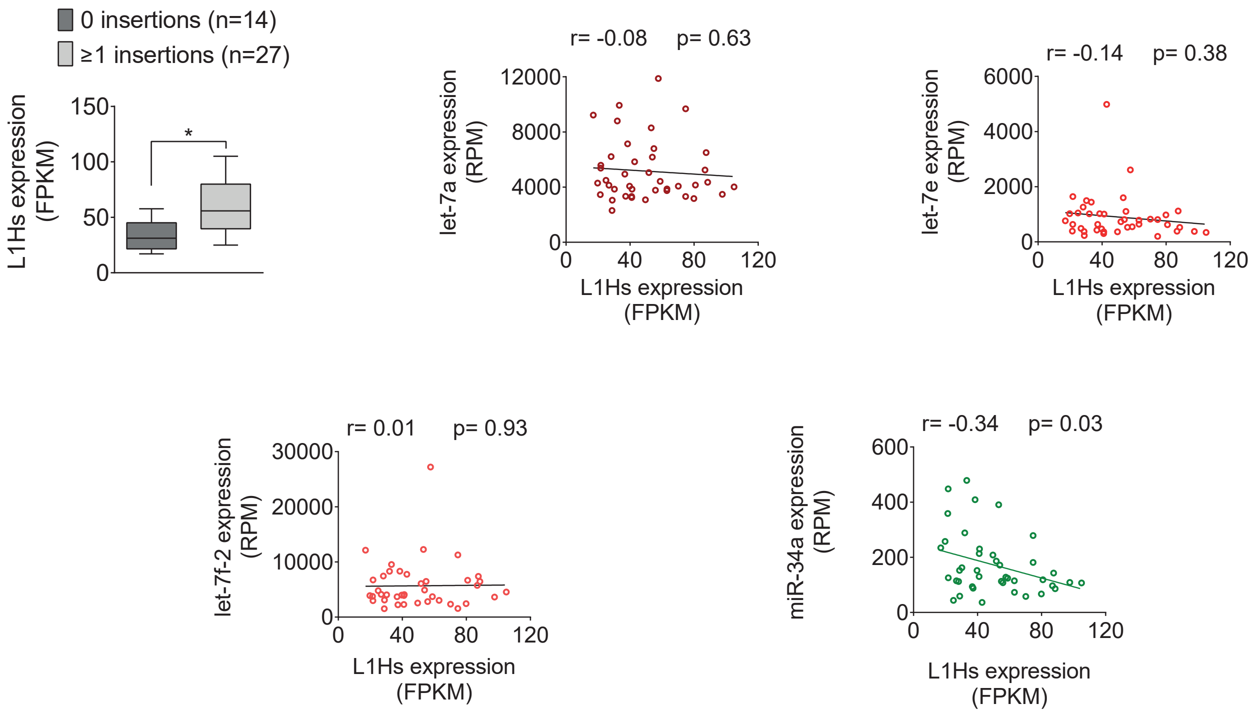

c

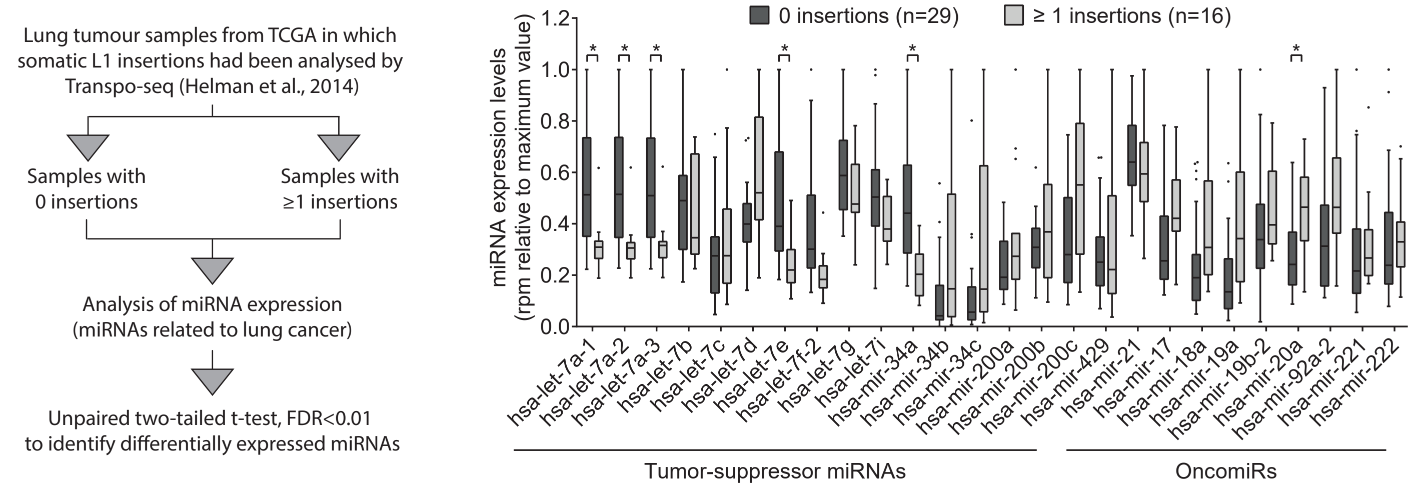

Supplementary Figure 1

d

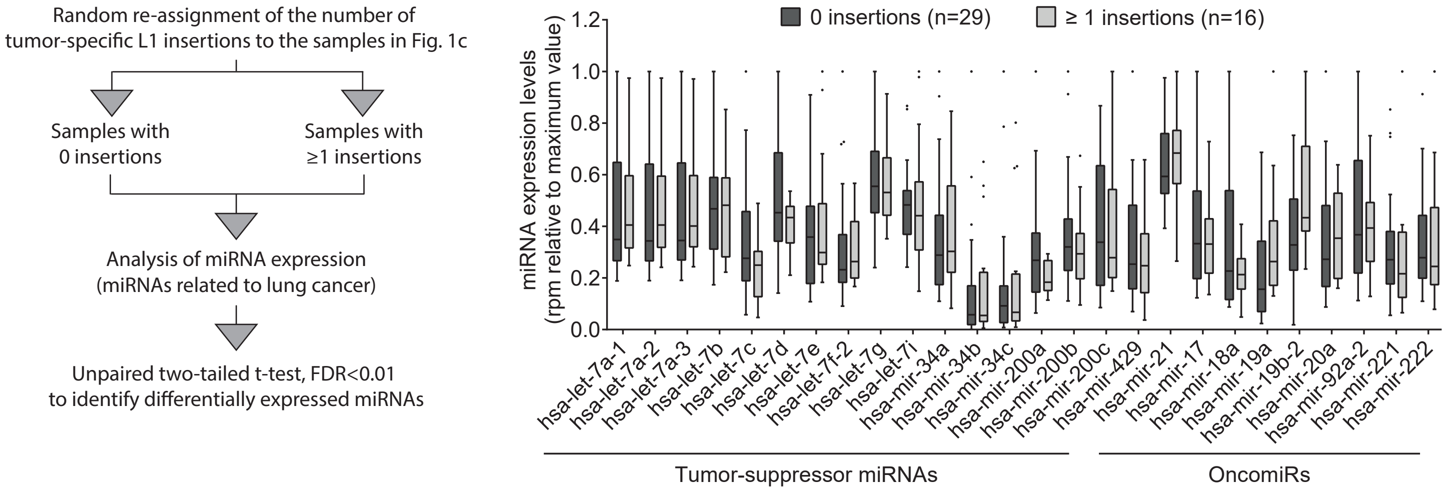

e

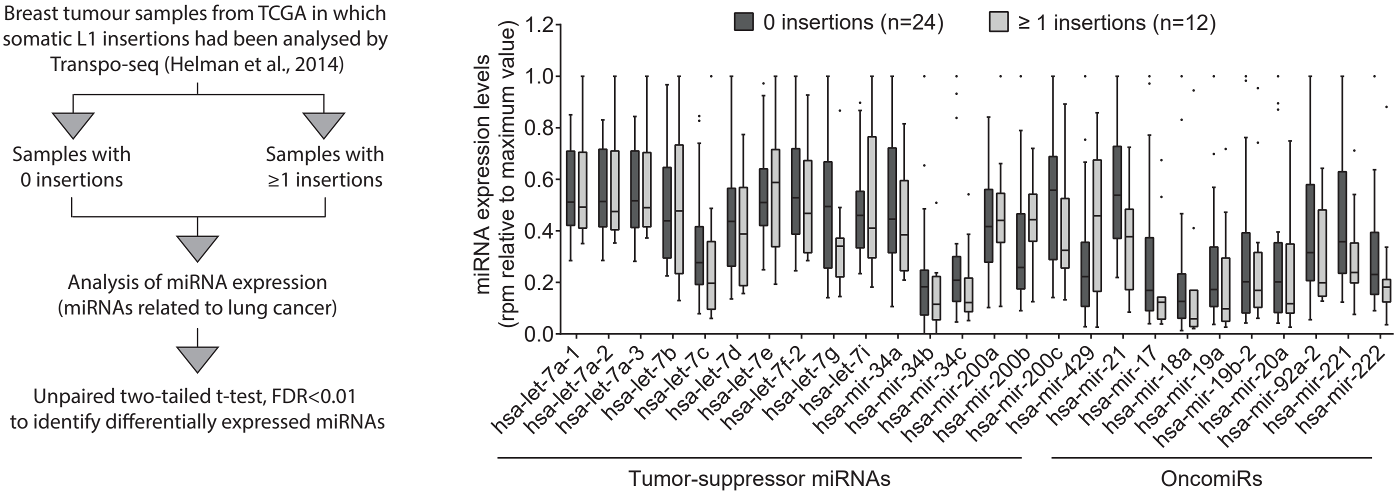

Supplementary Figure 1. Controls to corroborate the correlation between let-7 and miR-34a expression levels and accumulation of tumor-specific L1 insertions in lung tumor samples. (a) Analysis in Figure 1 was repeated after randomly re-assigning the value of tumor-specific L1 insertions to the samples, showing no significant correlation with miRNA levels. **(b)** RNA-seq analysis showed that L1Hs is overexpressed in lung tumor samples with tumor-specific L1 insertions (left panel). Pair-wise correlations between expression levels of L1Hs and let-7a, let-7e, let-7f-2 and miR-34a (Pearson' r).P-value was considered significant if <0.05. **(c)** Correlation between miRNA expression and tumor-specific L1 insertions identified by Helman and col. using Transpo-seq was analyzed as in Figures 1 and Supplementary 1a. Reduced expression of members of the let-7 family and miR-34a correlated with increased number of tumor-specific L1 insertions. **(d)** Analysis of Supplementary Fig 1c was repeated after randomly re-assigning the value of tumor-specific L1 insertions to the samples. **(e)** Analysis in (c) was repeated using breast samples, in which tumor-specific L1 insertions had been identified by Helman and col. using Transpo-seq. (a, c, d, e) A schematic representation of the bioinformatic analysis used is represented on a left panel. Differentially expressed miRNAs were identified applying an unpaired two-tailed t test adjusted by FDR<0.01. To enable representation of all miRNAs in one graph, expression (rpm) was relative to the maximum value of each miRNA in each case. Whiskers were calculated using the Tukey method. Individual black dots represent outliers. Boxes extend from 25th to 75th percentiles, and lines in the middle of the boxes represent the median.
Raw data are provided as a Source Data File.

Supplementary Figure 2

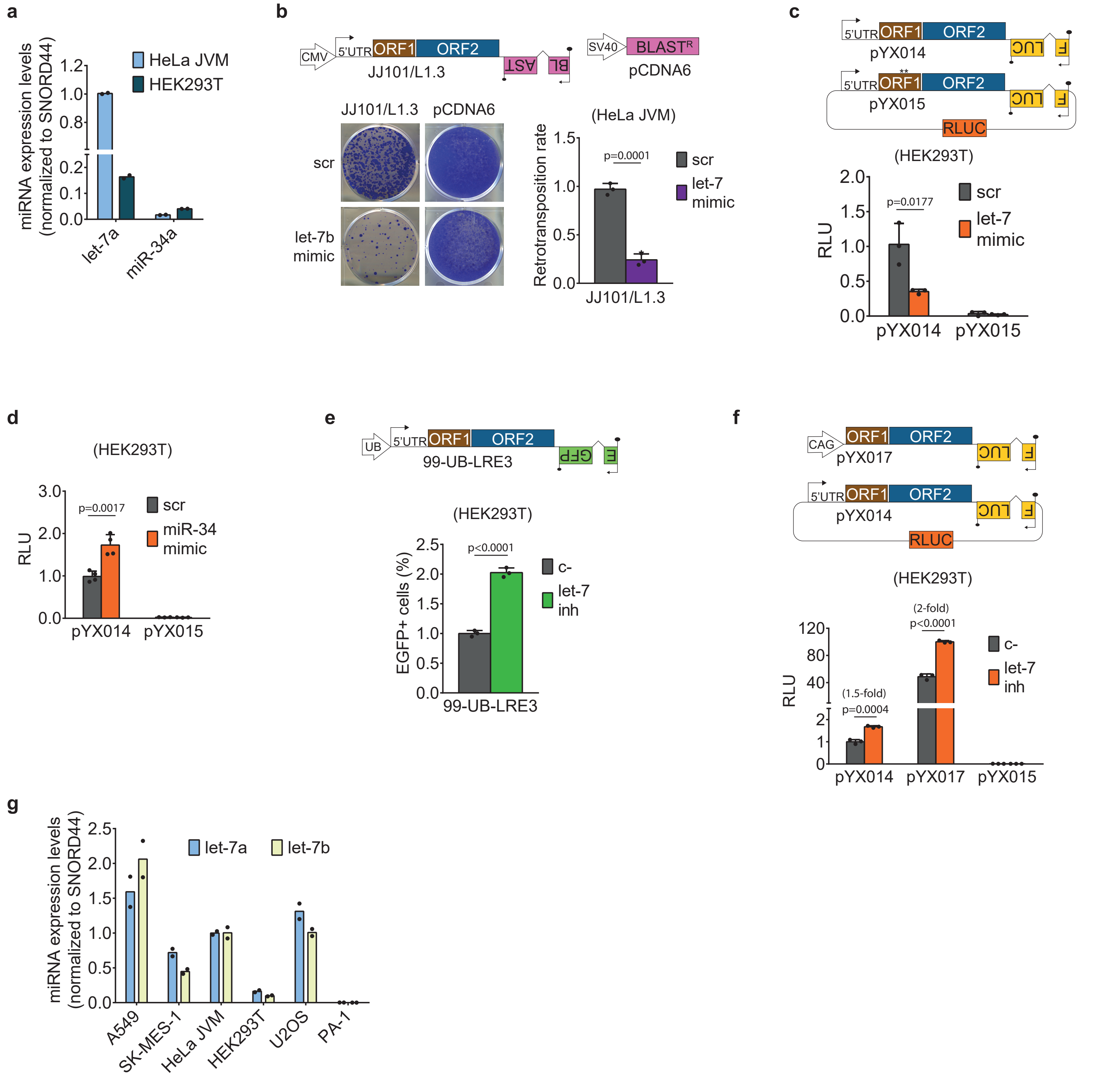

Supplementary Figure 2. Engineered human L1 retrotransposition rate increases and decreases upon let-7 depletion or overexpression, respectively, in a variety of cell lines. (a) RT-qPCR quantification of endogenous mature let-7a and mir-34a in HeLa and HEK293T cells. Average of two biological replicates is shown. **(b)** The structure of pJJ101/L1.3 is shown. HeLa cells were cotransfected with L1 plasmid and let-7b mimic or its control (scr). A representative well of three replicates is shown. Quantification is shown at the right as average of three replicates ± s.d. **(b-f)** Cell culture based retrotransposition assays with luciferase (c, d, f) or enhanced GFP (e) cassettes. In all cases, data is presented as mean value ± s.d. of at least three replicates. An unpaired two-tailed t-test was used to calculate p-values, and exact p-values are provided when p<0.05. In (c), (d) and (f), RLU: Relative Luminescence Units. **(c)** Structure of pYX014 and pYX015 are shown. HEK293T cells were cotransfected with one of them and let-7 mimic or its control (scr). Averages of three replicates are shown. **(d)** HEK293T cells were cotransfected with pYX014 or pYX015 and miR-34 mimic or its control (scr). Averages of four replicates are shown. **(e)** The structure of 99-UB-LRE3 is shown. HEK293T cells were cotransfected with L1 plasmid and let-7 inhibitor or its control (c-). Average of three replicates is shown. **(f)** Structure of pYX017 and pYX014 are shown. See (c) for pYX015 structure. HEK293T cells were cotransfected with L1 plasmid and let-7 inhibitor or its control (c-). Average of three replicates is shown. **(g)** RT-qPCR analysis of mature let-7a and let-7b miRNA expression levels in all cell lines used in this study. Average of two biological replicates is shown.
Raw data is provided as Source Data File

Supplementary Figure 3

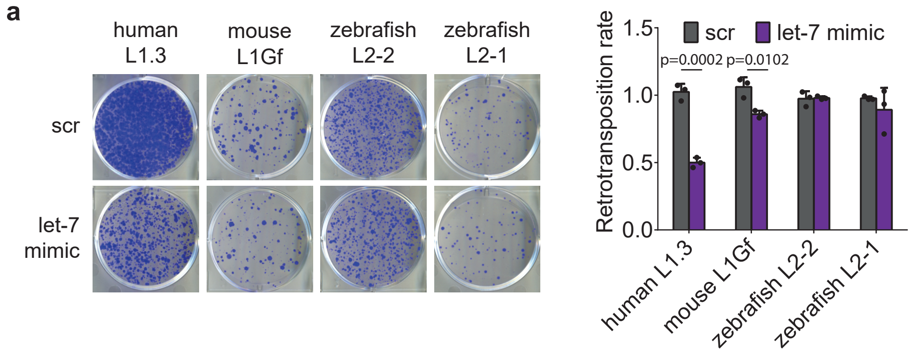

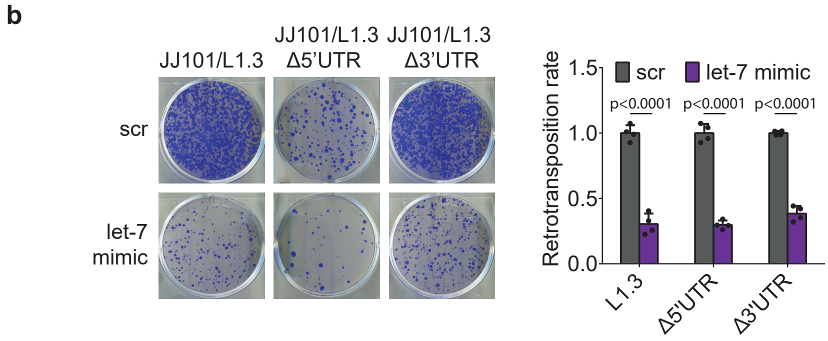

Supplementary Figure 3. Let-7 targets the coding sequence of human L1 mRNA. (a) Cell culture-based retrotransposition assay with *mneoI* tagged constructs. HeLa cells were co-transfected with LINEs from different species and let-7 mimic. For details of the constructs used see Fig. 3a. A representative well of three replicates is shown. Quantification is shown in the right as average of three replicates ± s.d. **(b)** Cell culture-based retrotransposition assay using *mblastI* tagged constructs. HeLa cells were co-transfected with LINEs lacking either the 5' or the 3' UTR (detailed structures shown in Fig. 3b) and let-7 mimic or its control (scr). A representative well of four replicates is shown. Quantification is shown in the right as average of four replicates ± s.d. **(a,b)** An unpaired two-tailed t-test was used to calculate p-values, and exact p-values are provided when p<0.05. Raw data is provided as Source Data File.

Supplementary Figure 4

a

```
GAAATTATAACAAACTATCTCT    bs1
    ||:|||  ||||:|||
TTGATATGTTGGATGATGGAGT    let-7a

CATCACACTACCTG--ACTTCA    bs2
 |:||||| ||||:  ||:|||
TTGGTGTGTTGGATGATGGAGT    let-7b
```

Predicted binding site	Location (Pos in L1Hs)	Prediction method/software	dG	Validated by siCHECK?
bs (1)	ORF2 (2650-2671)	mIRanda	-17.5	NO
bs (2)	ORF2 (4596-4616)	RNA22	-19.4	**YES**

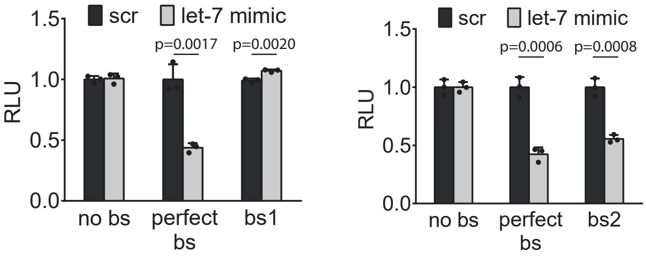

b

```
5'      GA G       G      3'   L1.3 (bs2rhmut)
   AGCUG  G AAUC AC
   UUGAU  U UUGG UG
3'      A  G    A  AUGGAGU 5'  let-7
```

Folding energy -9.7 Kcal/mol

(RNAhybrids)

c

```
L1PA1     AAAAAGAACAAAGCTGGAGGCATCACACTACCTGACTTCAAACTAT 4618
L1PA2     AAAAAGAACAAAGCTGGAGGCATCACACTACCTGACTTCAAACTAT 4619
L1PA3     AAAAAGAACAAAGCTGGAGGCATCACACTACCTGACTTCAAACTAT 4749
L1PA4     AAAAAGAACAAAGCTGGAGGCATCACACTACCTGACTTCAAACTAT 4746
L1PA5     AAAAAGAACAAAGCTGGAGGCATCACACTACCTGACTTCAAACTAT 4742
L1PA6     AAAAAGAACAAAGCTGGAGGCATCACGCTACCTGACTTCAAACTAT 4733
L1PA7     AAAAAGAACAAAGCTGGAGGCATCACGCTACCTGACTTCAAACTAT 5065
L1PA8     AAAAAGAACAAAGCTGGAGGCATCACGCTACCTGACTTCAAACTAT 5059
L1PA8A    AAAAAGAACAAAGCTGGAGGCATCACGCTACCTGACTTCAAACTAT 5047
L1PA10    AAAAAGAACAAAGCTGGAGGCATCACGCTACCCGACTTCAAACTAT 4987
L1PA11    AAAAAGAACAAAGCTGGAGGCATCACGCTACCTGACTTCAAACTAT 5118
L1PA12    AAAAAGAACAAAGCTGGAGGCATCACGCTACCCGACTTCAAACTAT 6380
L1PA13A   AAAAAGAACAAAGCTGGAGGCATCACGCTACCTGACTTCAAACTAT 5336
L1PA13B   AAAAAGAACAAAGCTGGAGGCATCACACTACCTGACTTCAAACTAT 5100
L1PA14    AAAAAGAACAAAGCTGGAGGCATCACATTACCTGACTTCAAACTAT 5896
L1PA15A   AAAAAGAACAAAGCTGGAGGCATCACATTACCTAACTTCAAACTAT 4796
L1PA15B   AAAAAGAACAAAGCTGGAGGCATCACATTACCTAACTTCAAACTAT 5203
L1PA16    AAAAAGAACAAAGCTGGAGGCATCACATTACCCGACTTCAAACTAT 5431
          **************************  ****   ************
```

d

```
Human_L1.3    AAGAACAAAGCTGGAGGCATCACACTACCTGACTTCAAACTATACTAC
Human_LRE3    AAGAACAAAGCTGGAGGCATCACACTACCTGACTTCAAACTATACTAC
Human_L1RP    AAGAACAAAGCTGGAGGCATCACACTACCTGACTTCAAACTATACTAC
Mouse_L1Gf    AAAAGAACCTCTGGTGGAATCACCATGCCTGACCTAAAGCTTTACTAC
              ** *  *   **** ** *****  * ****** * ** ** ******
```

```
Human_L1.3-ORF2p       ELEKTTLKFIWNQKRARIAKSILSQKNKAGGITLPDFKLYYKATVTKTAWYWYQNRDIDQ 898
Human_LRE3-ORF2p       ELEKTTLKFIWNQKRARIAKSILSQKNKAGGITLPDFKLYYKATVTKTAWYWYQNRDIDQ 898
Human_L1RP-ORF2p       ELEKTTLKFIWNQKRARIAKSILSQKNKAGGITLPDFKLYYKATVTKTAWYWYQNRDIDQ 898
Mouse_L1Gf-ORF2p       ELEGAICKFIWNNKKPRIAKTLLKDKRTSGGITMPDLKLYYRAIVIKTAWYWYRDRQVDQ 905
Zebrafish_L2.1-ORF2p   HITPILSSLHWLPVKFRIEFKIL--------------LLTYKALNNLAPVYLTNL----- 797
Zebrafish-L2.2-ORFp    HVTPLLVRLHWLPVAARIKFKTL--------------MFAYKVTSGLAPSYLHSL----- 893
                       .:       : *     **  . *                 : *:.     :   *
```

Supplementary Figure 4

Supplementary Figure 4. Identification of a let-7 binding site in human L1-ORF2. (a) The best predicted binding site of two different algorithms were tested with psiCHECK2. Location, method used for its prediction and ΔG of its predictive binding to L1.3 are summarized in the table. Predicted pairing of these sequences with let-7a and let-7b is shown on the left. Graphs below show the results of the psiCHECK2 assays with each of them (see Fig. 4b for a detailed structure of this vector). HEK293T cells were co-transfected with three different psiCHECK2 constructs and let-7 mimic or its control (scr). 'no bs' is a negative control (a sequence without complementarity to let-7) and 'perfect bs' is a positive control (with perfect complementarity to let-7). RLU: Relative Luciferase Units. Data is presented as average of three replicates ± s.d. An unpaired two-tailed t-test was used to calculate p-values, and exact p-values are provided when $p<0.05$. **(b)** RNAhybrid prediction of 'bs2rhmut' and let-7 interaction. Base-pairing between this region and let-7b is shown (green rectangle). Folding energy is shown on the right. **(c)** Alignment of the consensus sequence of L1PA1 to L1PA16 families showing conservation of the let-7 binding site ´bs2rh´ (in blue). **(d)** Alignment of the predicted binding site region in all L1s used in this study: human L1.3/L1RP/LRE3, mouse L1GF, and zebrafish L2.1 and L2.2. Alignment below was performed with ORF2 protein sequences to localize the binding site region within each LINE (asterisks denote conserved aminoacids). Human and mouse L1s nucleotide sequences were further analyzed. Blue nucleotides represent the 'bs2rh' region that was predicted by RNAhybrid and validated as a binding site in psiCHECK2. Red nucleotides are those which differ in mouse L1GF respect to human L1.3, LRE3 or L1RP.

Supplementary Figure 5

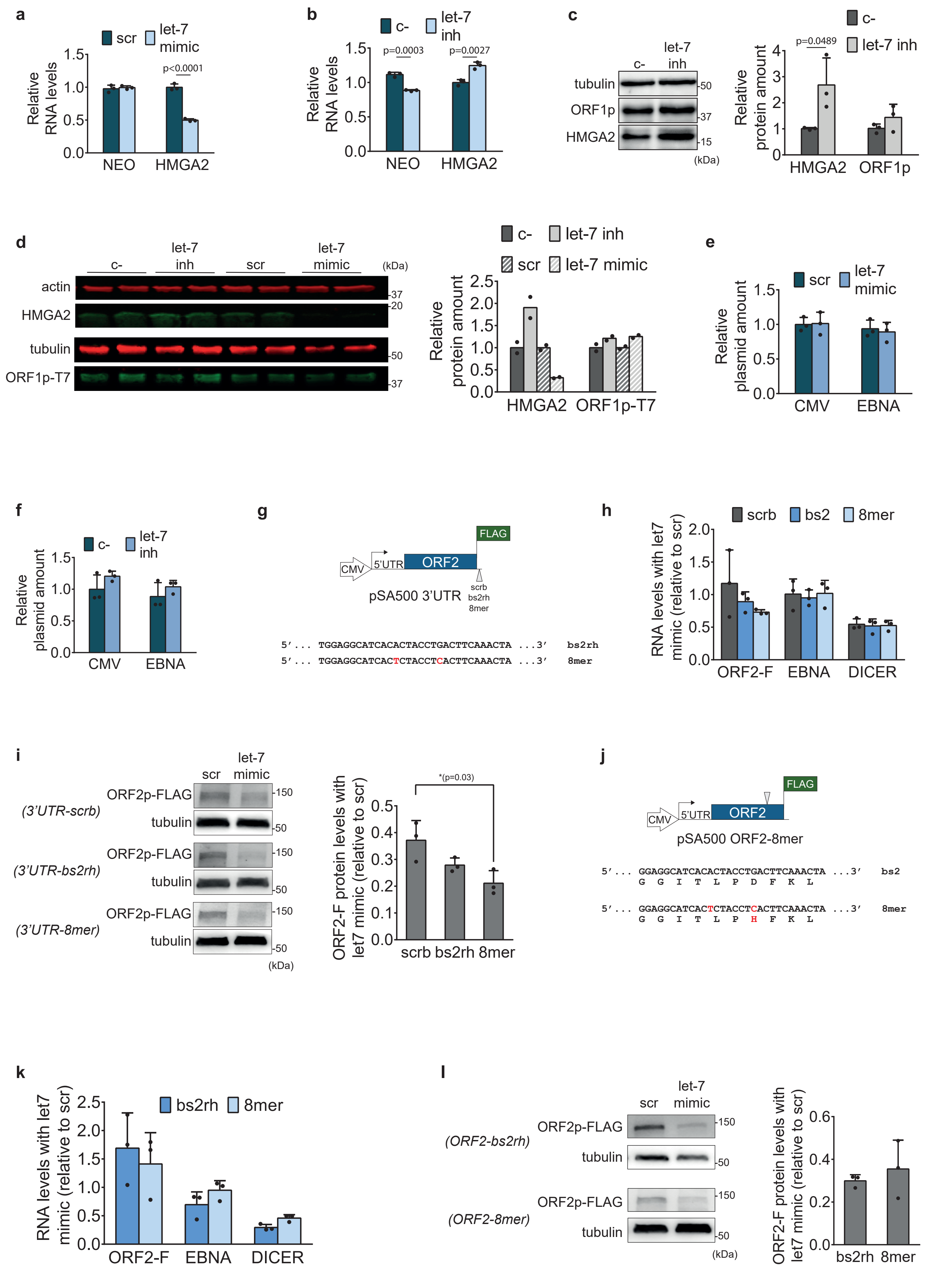

Supplementary Figure 5

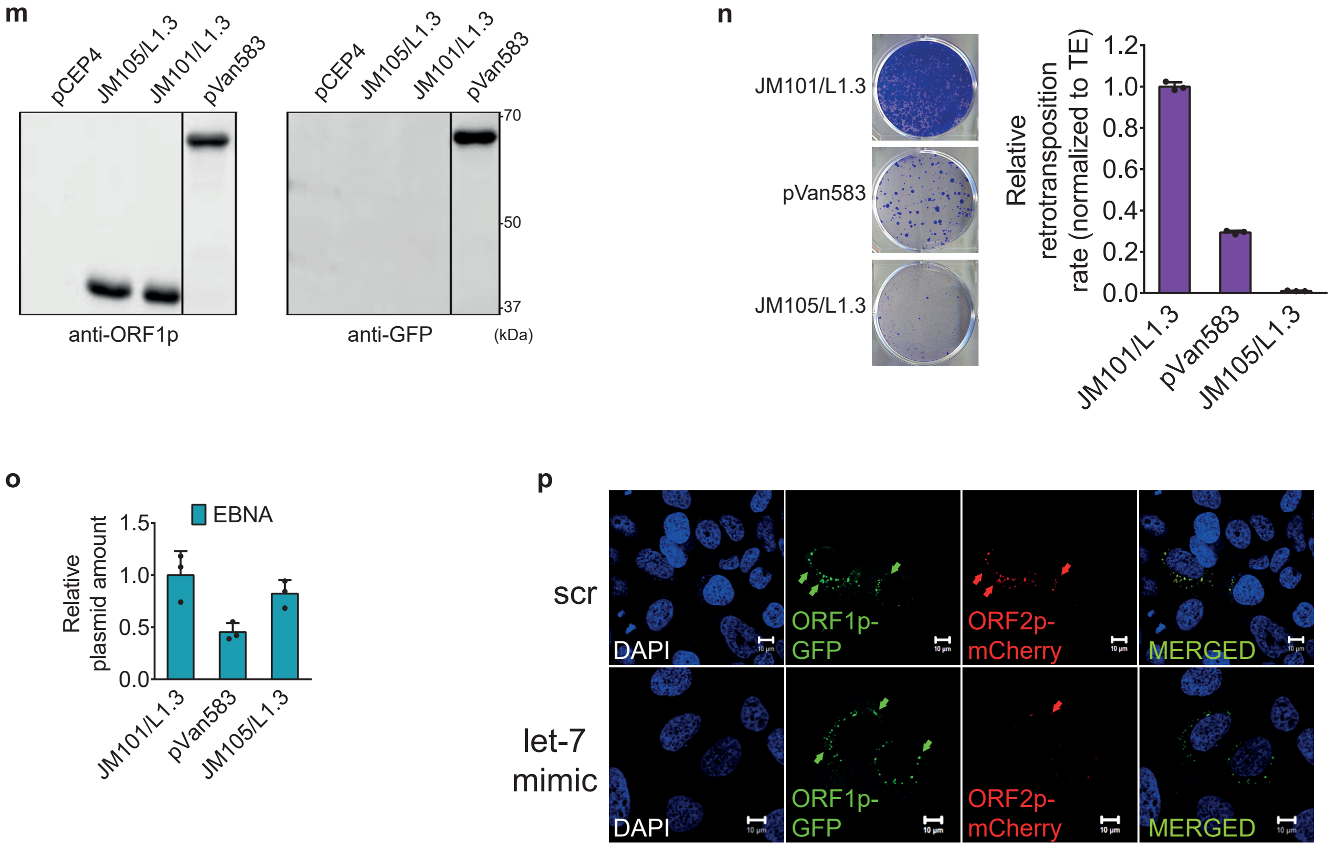

Supplementary Figure 5. Let-7 affects exogenous L1-ORF2p translation but not L1-ORF1p or L1 mRNA levels.
(a-b) RT-qPCR analysis of L1 mRNA levels transcribed from a plasmid. HEK293T were transfected with JM101/L1.3 and **(a)** let-7 mimic or **(b)** let-7 inhibitor. Exogenous L1 mRNA was specifically detected using primers against the spliced neomycin-resistance cassette. EBNA-1, expressed constitutively from the plasmid backbone, was used to normalize. HMGA2 normalized to GAPDH was used as a positive control. Data is presented as average of three replicates ± s.d. **(c)** Western blot analyses of endogenous L1-ORF1p and HMGA2 protein levels upon let-7 depletion. HEK293T cells were transfected with let-7 inhibitor or its control (c-). Data is presented as average of three replicates ± s.d. **(d)** Western blot analyses of stably-expressed T7-tagged L1-ORF1p upon depletion or overexpression of let-7. Stable Flp-In-293 cells expressing T7-tagged L1-ORF1p were transfected with let-7 inhibitor or mimic, or their controls (c- and scr, respectively). HMGA2 was used as a positive control. Western blot (left) and its quantification by Odyssey (right) are shown. Averages of two replicates are shown. **(e-f)** Transfection control for western blot shown in Fig. 5c. Plasmid levels (pSA500) upon let-7 overexpression (e) or depletion (f) were analyzed by qPCR using two different pairs of primers: CMV and EBNA. Genomic GAPDH was used to normalize. Data is presented as average of three replicates ± s.d. **(g)** Scheme of the different derivatives of pSA500 generated by insertion of different sequences in its 3'UTR: 'scrb', 'bs2rh' and '8mer'. Different nucleotides in 8mer compared to bs2rh are shown in red. **(h)** RT-qPCR analysis of ORF2-F, EBNA and DICER mRNA levels upon let-7 overexpression. HeLa cells were transfected with one of the three pSA500 3'UTR derivatives shown in (g) and let-7 mimic. Graph shows the RNA levels of the different mRNAs upon let-7 overexpression, relative to the scr condition, in each case. EBNA was used to normalize ORF2-F levels, and GAPDH was used to normalize EBNA and DICER. Data is presented as average of three replicates ± s.d.
(i) Western blot analyses of L1-ORF2p-FLAG upon let-7 overexpression. A fraction of cells from (h) were used. L1-ORF2p was detected using a FLAG antibody. Representative well and quantification of the western blot are shown. Data is presented as average of three replicates ± s.d. **(j)** Scheme of pSA500 ORF2-8mer generated by mutation of two nucleotides in the sequence of bs2rh. Different nucleotides and aminoacids in 8mer compared to bs2rh are shown in red. **(k)** RT-qPCR analysis of ORF2-F, EBNA and DICER mRNA levels upon let-7 overexpression. HeLa cells were transfected with pSA500 and pSA500ORF2-8mer showed in (j) and let-7 mimic. Graph shows the RNA levels of the different mRNAs upon let-7 overexpression, relative to the scr condition, in each case. EBNA was used to normalize ORF2-F levels, and GAPDH was used to normalize EBNA and DICER. Data is presented as average of three replicates ± s.d. **(l)** Western blot analyses of L1-ORF2p-FLAG upon let-7 overexpression. A fraction of cells from (k) were used. L1-ORF2p was detected using a FLAG antibody. Representative well and quantification of the western blot are shown. Data is presented as average of three replicates ± s.d. **(m)** Western blot analysis of ORF1p and ORF1p-GFP fusion in L1 RNP produced by transfection of L1-expressing plasmids in HEK-293T cells, and enriched by ultracentrifugation on sucrose cushion. pCEP4 represents the empty vector backbone and was used as a negative control. Membrane was first incubated with an anti-GFP antibody (3H9 clone), stripped, and then reprobed with anti-ORF1p antibody (SE-6798). One of two replicates is shown. **(n)** Cell culture-based retrotransposition assay comparing retrotransposition efficiency of different constructions. HeLa cells were transfected with either JM101/L1.3, pVan583 or JM105/L1.3 and selected with neomycin. Quantification (corrected for transfection efficiency, shown in (o)) is shown in the right. Data is presented as average of three replicates ± s.d. **(o)** Transfection efficiency comparison between JM101/L1.3 and pVan583. HeLa cells were transfected with one of the constructs, and plasmid levels were quantified by qPCR using EBNA primers and normalized to genomic GAPDH. Data is presented as average of three replicates ± s.d. **(n,o)** As negative control a mutant construct containing a missense mutation in the RT domain (D702A) was used (JM105/L1.3). **(p)** U2-OS cells were cotransfected with pVAN583 and let-7 mimic or its control (scr), and fluorescence was analyzed by confocal microscopy. Arrows indicate L1-ORF1p-EGFP or L1-ORF2p-mCherry foci. Images are representative of three (for scr) five (for let-7 mimic) replicates.
(a-c,e-l) An unpaired two-tailed t-test was used to calculate p-values, and exact p-values are provided when $p<0.05$.
Raw data are provided as a Source Data File.

Supplementary Figure 6

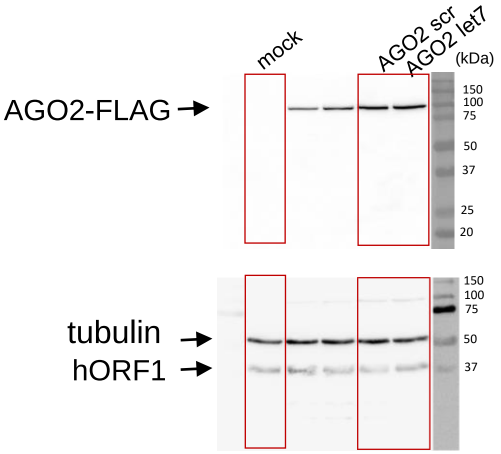

Input Fig 3d

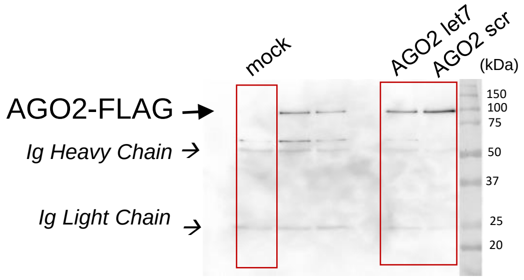

IP Fig 3d

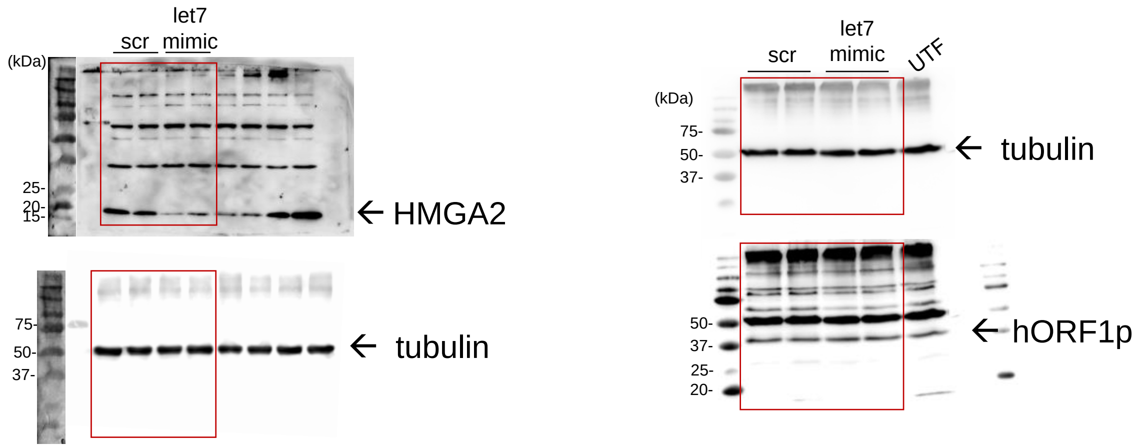

Fig 5b

Supplementary Figure 6. Uncropped versions of the western blots shown in this study.

Supplementary Figure 6

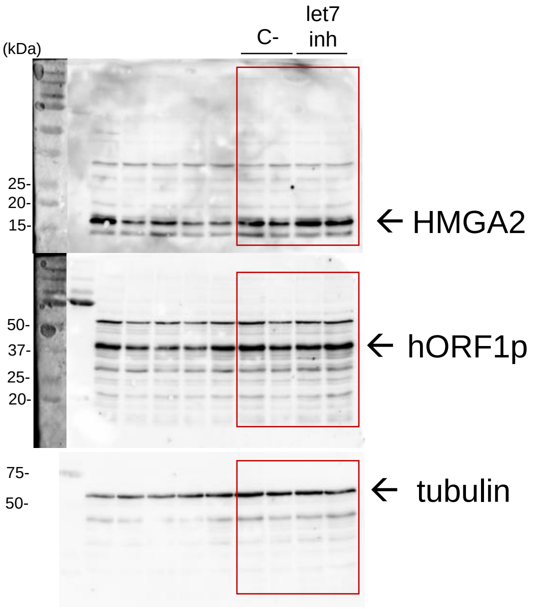

Supp Fig 5c

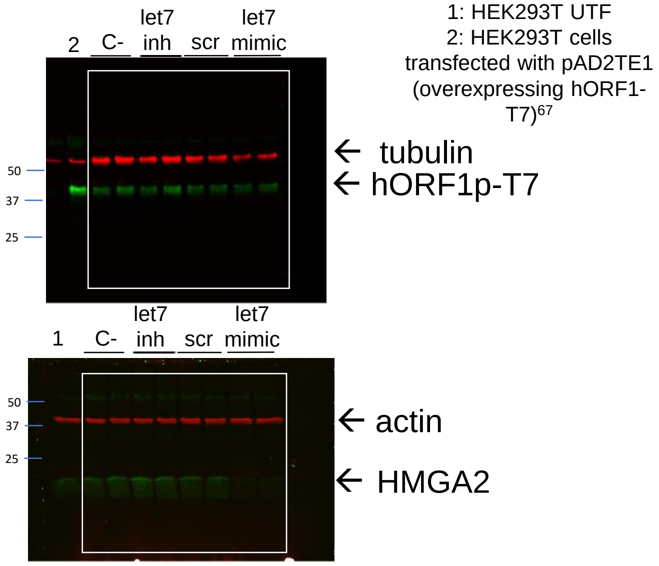

Supp Fig 5d

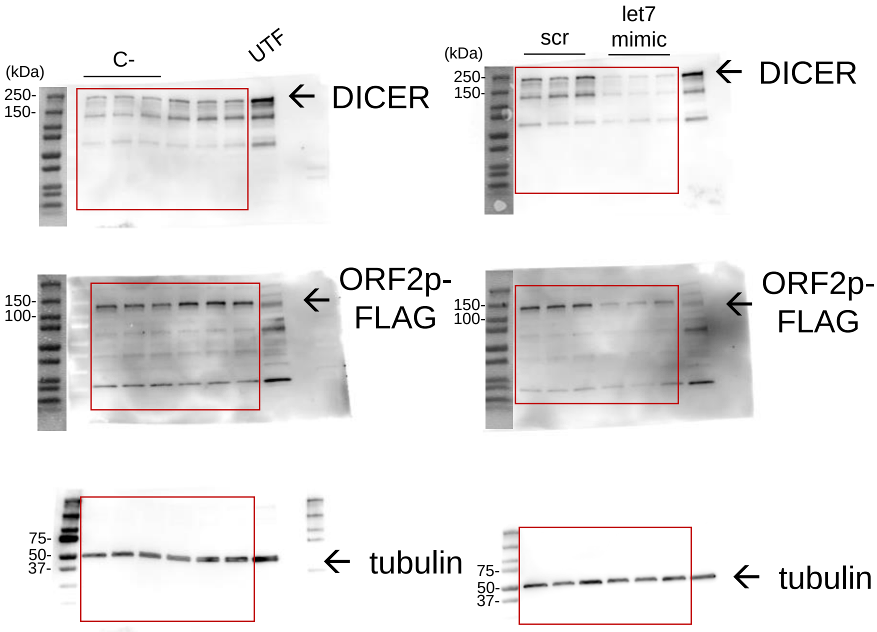

Fig 5c

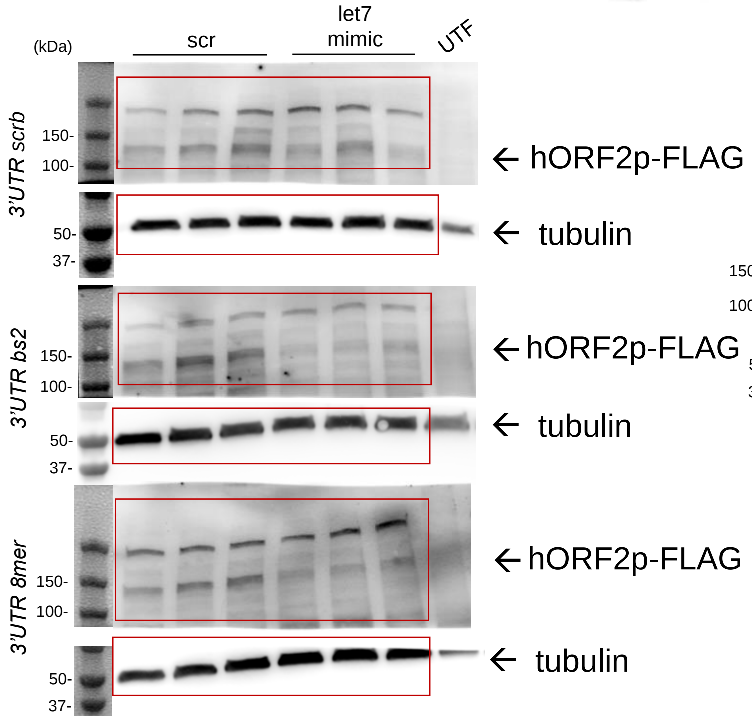

Supp Fig 5i

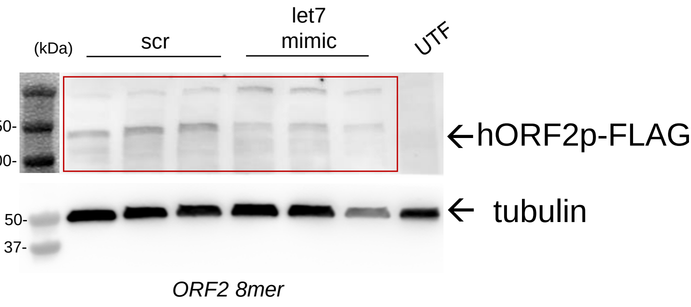

Supplementary Figure 6. Uncropped versions of the western blots shown in this study.

Sample	Types of tumor	Coverages		Unfiltered Poly L1			MELT insertion calls					
		PT	*NT*	*PT*	*NT*	*Common*	*PT*	*NT*	*NT-PT*	*Poly PT*	*Poly NT*	*Poly Common*
TCGA-18-3408	*LUSC*	70.3	34.5	138	136	98	9	0	0	48	43	43
TCGA-18-3415	*LUSC*	76.0	38.0	141	134	95	26	0	0	35	34	31
TCGA-18-4721	*LUSC*	69.5	44.6	138	141	102	16	0	1	56	57	54
TCGA-21-1076	*LUSC*	54.0	38.3	133	126	93	5	0	0	29	28	27
TCGA-21-1078	*LUSC*	44.4	45.3	118	120	78	0	1	0	14	15	14
TCGA-21-1083	*LUSC*	89.3	65.4	129	130	105	29	0	1	50	50	48
TCGA-22-5477	*LUSC*	56.0	44.2	126	131	94	17	0	0	45	47	42
TCGA-22-5485	*LUSC*	95.4	41.1	133	136	97	19	0	0	53	55	50
TCGA-22-5492	*LUSC*	81.0	32.8	136	140	103	20	0	0	45	47	42
TCGA-33-4586	*LUSC*	70.3	37.9	134	144	91	12	0	0	38	42	35
TCGA-34-2596	*LUSC*	51.8	45.6	213	136	84	55	0	1	37	41	32
TCGA-34-2600	*LUSC*	37.9	62.2	110	132	70	6	0	0	27	33	23
TCGA-43-3394	*LUSC*	47.2	46.4	138	147	108	5	0	0	26	30	23
TCGA-43-3920	*LUSC*	71.9	35.2	110	113	81	8	0	0	29	30	26
TCGA-56-1622	*LUSC*	44.3	41.9	111	124	78	3	0	0	17	25	16
TCGA-60-2698	*LUSC*	63.6	34.2	125	124	89	34	0	1	27	28	24
TCGA-60-2711	*LUSC*	47.1	52.9	143	137	99	5	0	0	29	28	27
TCGA-60-2713	*LUSC*	46.6	51.9	122	119	80	3	1	0	15	19	13
TCGA-60-2719	*LUSC*	62.5	29.0	125	123	90	4	0	0	11	12	9
TCGA-60-2724	*LUSC*	62.8	49.7	125	131	93	3	0	0	24	26	23
TCGA-66-2744	*LUSC*	66.3	51.2	130	128	99	4	0	0	38	36	36
TCGA-66-2759	*LUSC*	63.9	51.6	127	129	92	39	0	0	52	53	49
TCGA-66-2766	*LUSC*	46.8	44.1	133	125	82	9	0	0	15	11	11
TCGA-66-2789	*LUSC*	83.6	37.0	130	127	89	0	0	0	37	33	32
TCGA-66-2793	*LUSC*	73.9	35.4	129	143	91	54	0	0	42	43	41
TCGA-66-2795	*LUSC*	61.8	34.8	122	126	82	14	0	0	28	27	24
TCGA-38-4628	*LUAD*	84.5	34.6	125	121	75	0	0	0	13	12	10
TCGA-38-4630	*LUAD*	48.5	36.2	117	127	82	6	0	0	20	21	19
TCGA-49-4486	*LUAD*	34.7	35.3	106	113	70	0	0	0	10	11	10
TCGA-49-4510	*LUAD*	41.0	30.8	132	122	83	0	0	0	11	11	8
TCGA-49-4512	*LUAD*	55.8	40.5	125	126	92	0	0	0	31	28	27
TCGA-49-6742	*LUAD*	64.2	68.2	117	137	85	3	0	0	22	26	20
TCGA-50-5930	*LUAD*	46.3	45.6	127	122	87	0	0	0	24	23	23
TCGA-50-5932	*LUAD*	40.0	45.3	109	117	86	0	0	0	29	33	29
TCGA-50-6591	*LUAD*	45.1	51.2	114	131	80	0	0	0	22	26	21
TCGA-55-6972	*LUAD*	44.8	38.3	116	108	80	5	0	0	31	33	26
TCGA-55-6982	*LUAD*	64.8	33.6	139	138	92	0	0	0	21	16	16
TCGA-55-6984	*LUAD*	33.3	41.3	109	115	75	0	1	0	32	32	26
TCGA-55-6986	*LUAD*	39.7	33.4	131	130	89	0	0	0	20	20	17
TCGA-73-4659	*LUAD*	36.4	35.7	108	115	72	0	0	0	14	15	14
TCGA-91-6847	*LUAD*	46.2	42.6	101	125	68	0	0	1	22	30	19
TOTAL INSERTIONS							**413**	**3**	**5**	**1189**	**1230**	**1080**

		Description
**Sample:**		Case submitter ID in GDC database
**Type of tumor**		
**Coverage:**		Average depth of coverage in bam files
	PT	Primary tumor sample coverage
	NT	Normal tissue sample coverage
**Unfiltered Poly L1:**		Polymorphic L1 insertions found in MELT output before filtering
	PT	Number of primary tumor L1 polymorphic insertion calls
	NT	Number of normal tissue L1 polymorphic insertion calls
	Common	Number of L1 polymorphic insertion calls found both in tumor and normal tissue
**MELT insertion calls:**		L1 somatic insertion calls after filtering
	PT	Number of L1 insertion calls found in primary tumor
	NT	Number of L1 insertion calls found in normal tissue
	PT-NT	Number of L1 insertion calls found both in tumor and normal tissue
	Poly PT	Number of primary tumor L1 polymorphic insertion calls after filtering
	Poly NT	Number of normal tissue L1 polymorphic insertion calls after filtering
	Poly Common	Number of L1 polymorphic insertion calls found both in tumor and normal tissue after filtering

Supplementary Table 1. Summary of L1 insertions found by MELT.

	Discovery?	P value	Mean 0 insertions	Mean >1 insertions	Difference	SE of difference	t ratio	df
hsa-let-7a-1	*	< 0.0001	6389.63	4112.88	2276.76	508.865	4.47419	37
hsa-let-7a-2	*	< 0.0001	12754.3	8199.58	4554.68	1035.16	4.4	37
hsa-let-7a-3	*	< 0.0001	6448.99	4180.23	2268.76	514.737	4.40761	37
hsa-let-7b		0.700569	15437.2	14717.5	719.724	1857.49	0.387472	38
hsa-let-7c		0.337504	1257.44	1654.59	-397.152	408.714	0.971711	37
hsa-let-7d		0.0762467	690.492	897.586	-207.094	113.452	1.8254	36
hsa-let-7e	*	< 0.0001	1202.24	584.67	617.569	133.397	4.62955	37
hsa-let-7f-2	*	< 0.0001	7173.84	3887.56	3286.28	739.198	4.44574	37
hsa-let-7g		0.00515672	711.604	562.65	148.954	50.0942	2.97348	37
hsa-let-7i		0.175339	600.312	531.649	68.6635	49.6936	1.38174	37
hsa-mir-34a	*	< 0.0001	258.375	121.504	136.871	30.2276	4.52802	38
hsa-mir-34b		0.291543	14.1297	22.0518	-7.92218	7.4038	1.07002	37
hsa-mir-34c		0.0658459	40.1709	102.484	-62.3128	32.8725	1.89559	37
hsa-mir-200a		0.864283	1326.75	1388.6	-61.851	359.354	0.172117	37
hsa-mir-200b		0.602682	895.754	794.176	101.578	193.418	0.525174	36
hsa-mir-200c		0.538162	11985.1	13472.9	-1487.86	2393.83	0.621538	36
hsa-mir-429		0.0562723	353.184	195.655	157.529	79.863	1.97249	36
hsa-mir-21		0.51946	307470	290656	16814.2	25852.6	0.650389	37
hsa-mir-17		0.815847	837.164	882.253	-45.089	192.193	0.234603	36
hsa-mir-18a		0.00951988	12.7872	24.2019	-11.4146	4.16723	2.73914	36
hsa-mir-19a		0.0076794	24.9402	50.9265	-25.9862	9.21607	2.81966	37
hsa-mir-19b-2		0.332557	135.853	160.875	-25.0219	25.4848	0.981837	37
hsa-mir-20a		0.117302	212.595	298.805	-86.2105	53.7244	1.60468	36
hsa-mir-92a-2		0.294047	7299.51	9238.63	-1939.11	1821.07	1.06482	36
hsa-mir-221		0.769015	234.95	223.054	11.8957	40.1947	0.295953	35
hsa-mir-222		0.147941	94.1347	123.918	-29.7829	20.1298	1.47954	35

Supplementary Table 2. Multiple t-tests of lung cancer-related miRNA expression in samples with/without tumor-specific insertions (found by MELT). Unpaired two-tailed t-tests, adjusting by FDR<0.01, were performed to calculate statistical significance.

	Down-regulated NO INSERTIONS (adjusted p-value)	Up-regulated NO INSERTIONS (adjusted p-value)
hsa-let-7a-2	0.999670833493728	0.009919197811194
hsa-let-7a-1	0.999670833493728	0.011404852379541
hsa-let-7f-2	0.999670833493728	0.011404852379541
hsa-let-7a-3	0.999670833493728	0.022693746426405
hsa-let-7e	0.999670833493728	0.078094490857035
hsa-let-7g	0.9951132961285	0.872206669613474
hsa-mir-34a	0.9951132961285	0.872206669613474
hsa-mir-21	0.998834967282168	0.872206669613474
hsa-let-7b	0.772942472861957	0.95466322269098
hsa-let-7c	0.423601380637498	0.95466322269098
hsa-let-7d	0.423601380637498	0.95466322269098
hsa-let-7i	0.772942472861957	0.95466322269098
hsa-mir-34b	0.429905740326531	0.95466322269098
hsa-mir-34c	0.423601380637498	0.95466322269098
hsa-mir-200a	0.608911585117655	0.95466322269098
hsa-mir-200b	0.665217880956605	0.95466322269098
hsa-mir-200c	0.434085457694848	0.95466322269098
hsa-mir-429	0.639258429507727	0.95466322269098
hsa-mir-17	0.423601380637498	0.95466322269098
hsa-mir-18a	0.484865179133113	0.95466322269098
hsa-mir-19a	0.423601380637498	0.95466322269098
hsa-mir-19b-2	0.428260752603725	0.95466322269098
hsa-mir-20a	0.428260752603725	0.95466322269098
hsa-mir-92a-2	0.639258429507727	0.95466322269098
hsa-mir-221	0.428260752603725	0.95466322269098
hsa-mir-222	0.423601380637498	0.95466322269098

Supplementary Table 3. Rank-sum test of lung cancer-related miRNA expression in samples with/without tumor-specific insertions (found by MELT). Unpaired rank-sum test was performed to calculate significance. FDR-adjusted p-values are shown.

	Discovery?	P value	Mean 0 insertions	Mean >1 insertions	Difference	SE of difference	t ratio	df
hsa-let-7a-1		0.811259	0.480698	0.495713	-0.0150151	0.0624291	0.240515	37
hsa-let-7a-2		0.789314	0.469172	0.485841	-0.0166693	0.0619347	0.269144	37
hsa-let-7a-3		0.816877	0.48102	0.495479	-0.0144588	0.0619966	0.233219	37
hsa-let-7b		0.87881	0.584209	0.573199	0.01101	0.0717229	0.153508	38
hsa-let-7c		0.00640231	0.407236	0.213361	0.193875	0.0671812	2.88585	38
hsa-let-7d		0.27441	0.562443	0.484549	0.0778941	0.0701826	1.10988	36
hsa-let-7e		0.335279	0.341726	0.280913	0.060813	0.0622923	0.976252	37
hsa-let-7f-2		0.820621	0.416792	0.40006	0.0167322	0.0732697	0.228364	37
hsa-let-7g		0.45017	0.681717	0.630438	0.0512785	0.0672057	0.763008	38
hsa-let-7i		0.607081	0.563465	0.591099	-0.0276344	0.0532797	0.518667	37
hsa-mir-34a		0.648227	0.330608	0.366547	-0.0359388	0.0781491	0.459875	38
hsa-mir-34b		0.85133	0.244194	0.226401	0.0177933	0.0942753	0.188737	37
hsa-mir-34c		0.291671	0.269225	0.176315	0.09291	0.0868538	1.06973	37
hsa-mir-200a		0.0505448	0.414823	0.258322	0.156501	0.0774322	2.02114	37
hsa-mir-200b		0.0739224	0.43227	0.291067	0.141203	0.0767126	1.84068	36
hsa-mir-200c		0.020224	0.544989	0.367096	0.177893	0.0732138	2.42978	36
hsa-mir-429		0.00789936	0.382171	0.191162	0.191009	0.0677867	2.81779	35
hsa-mir-21		0.296335	0.693197	0.633363	0.0598333	0.0564844	1.05929	37
hsa-mir-17		0.437549	0.24329	0.2907	-0.0474101	0.0603897	0.78507	36
hsa-mir-18a		0.217464	0.371112	0.28663	0.0844819	0.0673011	1.25528	36
hsa-mir-19a		0.940266	0.34279	0.348968	-0.0061786	0.0818928	0.075447	37
hsa-mir-19b-2		0.821321	0.444056	0.461259	-0.0172033	0.0756331	0.227457	37
hsa-mir-20a		0.771917	0.384745	0.408165	-0.0234204	0.0801902	0.29206	36
hsa-mir-92a-2		0.703678	0.348132	0.377677	-0.0295447	0.0770598	0.383399	36
hsa-mir-221		0.0398273	0.463609	0.328545	0.135065	0.0632584	2.13513	35
hsa-mir-222		0.298158	0.402576	0.324532	0.0780442	0.0739587	1.05524	37

Supplementary Table 4. Multiple t-tests of lung cancer-related miRNA expression in samples after randomization of the number of insertions (found by MELT). Unpaired two-tailed t-tests. adjusting by FDR<0.01. were performed to calculate statistical significance.

	Discovery?	P value	Mean 0 insertions	Mean >1 insertions	Difference	SE of difference	t ratio	df
hsa-let-7a-1	*	< 0.0001	6389.63	4112.88	2276.76	508.865	4.47419	37
hsa-let-7a-2	*	< 0.0001	12754.3	8199.58	4554.68	1035.16	4.4	37
hsa-let-7a-3	*	< 0.0001	6448.99	4180.23	2268.76	514.737	4.40761	37
hsa-let-7b		0.700569	15437.2	14717.5	719.724	1857.49	0.387472	38
hsa-let-7c		0.337504	1257.44	1654.59	-397.152	408.714	0.971711	37
hsa-let-7d		0.0762467	690.492	897.586	-207.094	113.452	1.8254	36
hsa-let-7e	*	< 0.0001	1202.24	584.67	617.569	133.397	4.62955	37
hsa-let-7f-2	*	< 0.0001	7173.84	3887.56	3286.28	739.198	4.44574	37
hsa-let-7g		0.00515672	711.604	562.65	148.954	50.0942	2.97348	37
hsa-let-7i		0.175339	600.312	531.649	68.6635	49.6936	1.38174	37
hsa-mir-100		0.0308682	6353.47	3619.02	2734.45	1218.3	2.24448	37
hsa-mir-101-1		0.143803	11381.9	9328.17	2053.72	1375.16	1.49344	37
hsa-mir-103-1		0.345597	14274.3	16349.1	-2074.85	2171.02	0.955702	36
hsa-mir-106b		0.0270753	586.445	851.139	-264.695	114.994	2.30181	37
hsa-mir-10a		0.0143427	28188.1	20543.5	7644.66	2974.95	2.56967	37
hsa-mir-10b		0.821102	11268.3	12411.2	-1142.88	5017.36	0.227785	36
hsa-mir-1247		0.796181	18.0916	16.502	1.58957	6.1087	0.260214	36
hsa-mir-125a		0.0389207	829.867	596.371	233.496	109.055	2.14109	37
hsa-mir-125b-1		0.35568	530.09	443.163	86.9269	92.9365	0.935336	37
hsa-mir-126		0.698115	6045.68	5611.75	433.924	1110.07	0.390897	37
hsa-mir-128-1		0.0021347	64.9002	126.617	-61.7164	18.6523	3.30879	36
hsa-mir-128-2		0.00226531	50.6081	94.2402	-43.6321	13.2741	3.28701	36
hsa-mir-1307		0.111592	1452.57	1988.9	-536.335	329.256	1.62893	38
hsa-mir-140		0.109876	609.357	477.695	131.662	80.4261	1.63705	38
hsa-mir-141		0.0305471	2313.91	3802.62	-1488.71	661.921	2.24908	37
hsa-mir-142		0.00516305	3184.57	6203.09	-3018.52	1016.98	2.96812	38
hsa-mir-143		0.285539	58195.3	46484.4	11710.9	10802.6	1.08407	36
hsa-mir-145		0.299229	1448.57	1234.46	214.114	203.363	1.05287	37
hsa-mir-146b		0.545521	996.727	1113.16	-116.43	190.836	0.610105	37
hsa-mir-148a		0.161206	61209.2	79392.5	-18183.2	12732	1.42816	39
hsa-mir-148b		0.0384939	267.314	365.963	-98.6481	45.9197	2.14828	36
hsa-mir-152		0.012338	282.767	542.564	-259.797	98.4552	2.63873	35
hsa-mir-155		0.0602033	323.66	484.118	-160.458	82.7688	1.93863	37
hsa-mir-16-1	*	0.00096899	830.11	1434.27	-604.164	168.916	3.57672	38
hsa-mir-17		0.815847	837.164	882.253	-45.089	192.193	0.234603	36
hsa-mir-181a-1	*	< 0.0001	2348.29	1221.24	1127.06	240.133	4.69347	37
hsa-mir-181a-2		0.323303	1000.45	1157.06	-156.611	156.387	1.00143	36
hsa-mir-181b-1	*	0.00011176	633.475	276.068	357.406	82.4227	4.33626	36
hsa-mir-182		0.835261	35813.4	34373.7	1439.75	6873.23	0.209472	36
hsa-mir-183		0.0268516	13969	24303.7	-10334.7	4472.2	2.31088	35
hsa-mir-186		0.0517296	348.563	474.994	-126.431	62.8911	2.01032	37
hsa-mir-18a		0.00951988	12.7872	24.2019	-11.4146	4.16723	2.73914	36
hsa-mir-191		0.00430778	807.206	570.062	237.144	77.8223	3.04725	36
hsa-mir-192		0.383406	600.689	1471.79	-871.105	987.182	0.882416	36
hsa-mir-199a-1		0.655153	1438.37	1583.45	-145.085	322.227	0.450257	37
hsa-mir-199a-2		0.444745	2366.91	2801.86	-434.948	563.065	0.772466	37
hsa-mir-199b		0.243662	2893.91	3769.63	-875.726	739.151	1.18477	37
hsa-mir-19a		0.0076794	24.9402	50.9265	-25.9862	9.21607	2.81966	37
hsa-mir-19b-2		0.332557	135.853	160.875	-25.0219	25.4848	0.981837	37
hsa-mir-200a		0.545175	1641.77	1388.6	253.172	414.706	0.610485	38
hsa-mir-200b		0.203681	1111.96	794.176	317.786	245.582	1.29401	37
hsa-mir-200c		0.538162	11985.1	13472.9	-1487.86	2393.83	0.621538	36
hsa-mir-203		0.0158299	11718.8	32772.2	-21053.4	8324.57	2.52907	37
hsa-mir-20a		0.117302	212.595	298.805	-86.2105	53.7244	1.60468	36
hsa-mir-21		0.51946	307470	290656	16814.2	25852.6	0.650389	37
hsa-mir-210		0.0172087	1947.49	3770.29	-1822.8	730.746	2.49443	37
hsa-mir-22		0.895489	75762.6	74727.7	1034.97	7824.83	0.132268	37
hsa-mir-221		0.769015	234.95	223.054	11.8957	40.1947	0.295953	35
hsa-mir-222		0.147941	94.1347	123.918	-29.7829	20.1298	1.47954	35
hsa-mir-23a		0.00199196	3344.79	5408.62	-2063.83	622.717	3.31423	39
hsa-mir-23b		0.00407049	1654.61	2647.63	-993.021	323.595	3.06871	36
hsa-mir-24-2		0.0272395	2236.27	3016.98	-780.713	339.172	2.30182	36
hsa-mir-25		0.00378113	6370.21	12381.6	-6011.35	1941.31	3.09654	36
hsa-mir-26a-2		0.00253955	2939.4	1919.34	1020.06	314.355	3.24494	36
hsa-mir-26b		0.0544779	1172.98	917.991	254.989	128.503	1.98431	38
hsa-mir-27a		0.137614	1486.71	1963.04	-476.33	313.678	1.51853	36
hsa-mir-27b		0.346726	2006.64	2317.66	-311.022	326.327	0.953098	37
hsa-mir-28		0.2459	4913.7	5658.42	-744.722	631.623	1.17906	37
hsa-mir-29a	*	< 0.0001	12806.3	4979.3	7827.01	1627.22	4.81005	38
hsa-mir-29b-1		0.00373056	632.847	353.857	278.991	90.2772	3.09038	38
hsa-mir-29b-2		0.00271523	684.27	379.423	304.847	95.0333	3.20779	38
hsa-mir-29c		0.00780712	2796.16	1535.74	1260.42	448.71	2.80898	38
hsa-mir-30a		0.435523	20155.8	17960.5	2195.26	2785.49	0.788107	38
hsa-mir-30b		0.00191122	711.898	415.752	296.146	88.4233	3.34919	36
hsa-mir-30c-2		0.213525	526.853	623.005	-96.152	75.9661	1.26572	37
hsa-mir-30d		0.00791168	9850.62	6235.47	3615.15	1285.41	2.81245	36
hsa-mir-30e		0.173315	15094.5	17274.6	-2180.11	1569.36	1.38917	36
hsa-mir-34a	*	< 0.0001	258.375	121.504	136.871	30.2276	4.52802	38
hsa-mir-34b		0.291543	14.1297	22.0518	-7.92218	7.4038	1.07002	37
hsa-mir-34c		0.0658459	40.1709	102.484	-62.3128	32.8725	1.89559	37
hsa-mir-361		0.0210504	384.11	283.146	100.964	41.8974	2.4098	37
hsa-mir-374a		0.417523	875.585	954.074	-78.4886	95.7578	0.819657	38
hsa-mir-375	*	0.00092376	21228.1	3623.56	17604.6	4887.95	3.60163	37
hsa-mir-429		0.0562723	353.184	195.655	157.529	79.863	1.97249	36
hsa-mir-532		0.11409	698.079	927.46	-229.381	141.741	1.61831	37
hsa-mir-92a-2		0.294047	7299.51	9238.63	-1939.11	1821.07	1.06482	36
hsa-mir-93		0.0120629	3794.53	6767.78	-2973.25	1124.56	2.64392	36
hsa-mir-99b		0.265917	37791.9	31273.6	6518.25	5770.44	1.12959	37
hsa-mir-99b		0.701176	27724	29320.3	-1596.38	4127.95	0.386724	37
hsa-mir-99b		0.34135	30202.9	26313.6	3889.3	4040.07	0.962683	41

Supplementary Table 5. Multiple t-tests of miRNA expression in samples with/without tumor-specific insertions (found by MELT). including all miRNAs expressed in lung cancer. Unpaired two-tailed t-tests, adjusting by FDR<0.01, were performed to calculate statistical significance.

	Discovery?	P value	Mean 0 insertions	Mean >1 insertions	Difference	SE of difference	t ratio	df
hsa-let-7a-1	*	0.0010389	6649.04	3861.02	2788.03	789.586	3.531	41
hsa-let-7a-2	*	0.00103993	13280.8	7674.08	5606.71	1588.01	3.53066	41
hsa-let-7a-3	*	0.00119799	6711.58	3936.7	2774.88	797.016	3.48159	41
hsa-let-7b		0.459978	16965.3	15325	1640.27	2198.5	0.746085	40
hsa-let-7c		0.252202	1359.77	1733.47	-373.697	321.769	1.16139	41
hsa-let-7d		0.00400422	628.825	895.523	-266.698	87.5773	3.04528	42
hsa-let-7e	*	0.00080871	1011.89	520.1	491.788	135.959	3.61717	41
hsa-let-7f-2		0.00451808	6594.19	3489.51	3104.68	1030.17	3.01375	39
hsa-let-7g		0.037565	703.605	572.997	130.608	60.817	2.14756	42
hsa-let-7i		0.0897111	618.008	491.085	126.923	72.9825	1.73909	40
hsa-mir-34a	*	0.00013916	247.358	106.943	140.415	33.3277	4.21318	40
hsa-mir-34b		0.0383327	12.3378	28.8924	-16.5546	7.73492	2.14025	41
hsa-mir-34c		0.0360055	46.9052	110.132	-63.227	29.1619	2.16813	41
hsa-mir-200a		0.0779602	1035.25	1478.11	-442.86	244.957	1.80791	41
hsa-mir-200b		0.152301	696.547	900.843	-204.296	139.999	1.45927	40
hsa-mir-200c		0.0046207	9935.68	16573.6	-6637.9	2215.26	2.99644	41
hsa-mir-429		0.43293	178.86	211.756	-32.8956	41.5255	0.792179	40
hsa-mir-21		0.320546	329966	302639	27326.7	27176.8	1.00552	41
hsa-mir-17		0.0236508	633.971	932.884	-298.913	127.18	2.35031	41
hsa-mir-18a		0.0193843	15.7268	26.9796	-11.2528	4.62348	2.43382	41
hsa-mir-19a		0.00416747	23.5588	46.7878	-23.229	7.64293	3.03928	40
hsa-mir-19b-2		0.181101	108.289	135.447	-27.1586	19.9443	1.36172	39
hsa-mir-20a	*	0.00223585	177.453	300.195	-122.742	37.5713	3.2669	40
hsa-mir-92a-2		0.0399419	5822.92	8259.04	-2436.12	1148.13	2.12182	41
hsa-mir-221		0.711901	235.589	255.839	-20.2506	54.4554	0.371875	41
hsa-mir-222		0.677125	106.974	116.9	-9.92579	23.6633	0.419459	40

Supplementary Table 6. Multiple t-tests of lung cancer-related miRNA expression in samples with/without tumor-specific insertions (found by Transpo-Seq). Unpaired two-tailed t-tests, adjusting by FDR<0.01, were performed to calculate statistical significance.

	Discovery?	P value	Mean 0 insertions	Mean >1 insertions	Difference	SE of difference	t ratio	df
hsa-let-7a-1	*	0.0010389	6649.04	3861.02	2788.03	789.586	3.531	41
hsa-let-7a-2	*	0.00103993	13280.8	7674.08	5606.71	1588.01	3.53066	41
hsa-let-7a-3	*	0.00119799	6711.58	3936.7	2774.88	797.016	3.48159	41
hsa-let-7b		0.459978	16965.3	15325	1640.27	2198.5	0.746085	40
hsa-let-7c		0.252202	1359.77	1733.47	-373.697	321.769	1.16139	41
hsa-let-7d		0.00400422	628.825	895.523	-266.698	87.5773	3.04528	42
hsa-let-7e	*	0.00080871	1011.89	520.1	491.788	135.959	3.61717	41
hsa-let-7f-2		0.00451808	6594.19	3489.51	3104.68	1030.17	3.01375	39
hsa-let-7g		0.037565	703.605	572.997	130.608	60.817	2.14756	42
hsa-let-7i		0.0897111	618.008	491.085	126.923	72.9825	1.73909	40
hsa-mir-100		0.119421	6957.06	3528.24	3428.82	2154.76	1.59128	40
hsa-mir-101-1		0.0111306	12970.7	8765.45	4205.22	1579.69	2.66205	40
hsa-mir-103-1		0.0227826	11917.9	16054.6	-4136.74	1748.31	2.36613	41
hsa-mir-106b	*	0.00041307	554.632	935.879	-381.247	99.1694	3.8444	41
hsa-mir-10a		0.555026	22781.2	20612.4	2168.74	3643.37	0.595255	40
hsa-mir-10b		0.249851	8534.46	12088.6	-3554.18	3044.9	1.16726	41
hsa-mir-1247		0.793903	18.0602	16.3879	1.67225	6.35844	0.262997	40
hsa-mir-125a		0.0718557	620.823	489.48	131.343	71.0811	1.84779	41
hsa-mir-125b-1		0.523782	408.383	346.686	61.6967	95.9255	0.643174	40
hsa-mir-126		0.340332	4188.06	5023.98	-835.918	866.216	0.965023	40
hsa-mir-128-1	*	0.00087143	66.7548	129.475	-62.7207	17.4292	3.59859	40
hsa-mir-128-2	*	0.00015327	47.5223	96.1291	-48.6068	11.5932	4.19269	39
hsa-mir-1307		0.00254383	1228.86	2080.89	-852.034	265.008	3.21512	41
hsa-mir-140		0.142376	585.044	465.254	119.791	80.0871	1.49576	41
hsa-mir-141		0.00718043	2461.99	3893.56	-1431.57	505.895	2.82978	41
hsa-mir-142		0.565181	4554.63	5139.64	-585.008	1008.88	0.579862	41
hsa-mir-143		0.023165	69682.2	42261.3	27420.9	11623.5	2.3591	41
hsa-mir-145		0.267181	1237.3	1028.65	208.648	185.42	1.12527	40
hsa-mir-146b		0.0820425	1494.59	1074.8	419.793	235.759	1.78061	43
hsa-mir-148a		0.152025	84403.9	65727.2	18676.6	12807.1	1.4583	43
hsa-mir-148b		0.467341	328.614	369.769	-41.155	56.0836	0.733815	40
hsa-mir-151a		0.459035	2993.58	3238.22	-244.637	327.12	0.747852	39
hsa-mir-152		0.0639914	331.083	458.198	-127.115	66.7281	1.90497	40
hsa-mir-155		0.130193	341.27	449.531	-108.26	70.0283	1.54595	39
hsa-mir-16-1		0.0736175	958.08	1238.03	-279.951	152.479	1.836	41
hsa-mir-17		0.0236508	633.971	932.884	-298.913	127.18	2.35031	41
hsa-mir-18a		0.0193843	15.7268	26.9796	-11.2528	4.62348	2.43382	41
hsa-mir-181a-1		0.00537774	2042.15	1319.19	722.96	245.594	2.94372	40
hsa-mir-181a-2		0.906397	1125.88	1107.3	18.5828	157.04	0.118332	40
hsa-mir-181b-1	*	0.00072474	581.012	321.778	259.234	70.9332	3.65463	41
hsa-mir-182		0.0841456	27737.5	36189.3	-8451.82	4774.75	1.77011	41
hsa-mir-183	*	< 0.0001	12057.1	24025.6	-11968.5	2717.66	4.40398	41
hsa-mir-186		0.239244	382.762	458.933	-76.1709	63.7813	1.19425	41
hsa-mir-19a		0.00416747	23.5588	46.7878	-23.229	7.64293	3.03928	40
hsa-mir-19b-2		0.181101	108.289	135.447	-27.1586	19.9443	1.36172	39
hsa-mir-191		0.191718	724.401	576.374	148.027	111.516	1.32741	41
hsa-mir-192		0.803246	838.475	905.982	-67.5067	269.11	0.250852	39
hsa-mir-199a-1		0.598369	1303.68	1451.13	-147.45	277.695	0.530979	40
hsa-mir-199a-2		0.581062	2277.46	2554.76	-277.308	498.43	0.556363	40
hsa-mir-199b		0.605234	3043.83	3402.08	-358.256	687.621	0.521008	40
hsa-mir-20a	*	0.00223585	177.453	300.195	-122.742	37.5713	3.2669	40
hsa-mir-200a		0.0779602	1035.25	1478.11	-442.86	244.957	1.80791	41
hsa-mir-200b		0.152301	696.547	900.843	-204.296	139.999	1.45927	40
hsa-mir-200c		0.0046207	9935.68	16573.6	-6637.9	2215.26	2.99644	41
hsa-mir-203	*	0.00201005	9763.98	30921.1	-21157.2	6401.4	3.30508	40
hsa-mir-21		0.320546	329966	302639	27326.7	27176.8	1.00552	41
hsa-mir-210	*	0.00074486	1540.32	3259.13	-1718.81	471.515	3.64529	41
hsa-mir-22		0.266497	64120.7	71701.1	-7580.31	6728.99	1.12652	41
hsa-mir-221		0.711901	235.589	255.839	-20.2506	54.4554	0.371875	41
hsa-mir-222		0.677125	106.974	116.9	-9.92579	23.6633	0.419459	40
hsa-mir-23a		0.00290393	3738.53	5197.75	-1459.22	461.418	3.16248	42
hsa-mir-23b		0.0844826	2083.79	2694.8	-611.002	345.567	1.76811	41
hsa-mir-24-2		0.632854	2617.8	2810.27	-192.466	399.879	0.481311	41
hsa-mir-25	*	< 0.0001	6493.3	14823.1	-8329.81	1705.08	4.88529	41
hsa-mir-26a-2	*	0.00042198	3311.49	1779.25	1532.24	399.306	3.83726	41
hsa-mir-26b		0.341009	955.44	828.891	126.549	131.361	0.963372	41
hsa-mir-27a		0.877617	1709.25	1664.89	44.3625	286.248	0.154979	40
hsa-mir-27b		0.348142	2458.61	2079.79	378.828	399.265	0.948815	42
hsa-mir-28		0.0853609	4880.43	6318.45	-1438.02	816.168	1.76192	42
hsa-mir-29a	*	< 0.0001	11772.1	4468.46	7303.65	1575.09	4.63696	41
hsa-mir-29b-1	*	0.00102067	584.082	317.214	266.869	75.448	3.53712	41
hsa-mir-29b-2	*	0.00125344	626.645	360.234	266.411	77.0026	3.45977	42
hsa-mir-29c	*	0.00048511	2710.04	1343.48	1366.56	359.751	3.79863	40
hsa-mir-30a		0.98941	17710.5	17669.1	41.4525	3103.71	0.0133558	40
hsa-mir-30b		0.011888	903.381	409.363	494.018	187.644	2.63274	41
hsa-mir-30c-2		0.589226	540.557	577.704	-37.1467	68.241	0.544345	40
hsa-mir-30d		0.0458337	11205.3	6985.72	4219.61	2045.56	2.06282	39
hsa-mir-30e		0.562658	17124.9	16152	972.928	1666.65	0.583762	40
hsa-mir-34a	*	0.00013916	247.358	106.943	140.415	33.3277	4.21318	40
hsa-mir-34b		0.0383327	12.3378	28.8924	-16.5546	7.73492	2.14025	41
hsa-mir-34c		0.0360055	46.9052	110.132	-63.227	29.1619	2.16813	41
hsa-mir-361		0.269269	318.97	271.97	47	41.9532	1.1203	40
hsa-mir-374a		0.804948	973.355	948.577	24.7778	99.672	0.248593	40
hsa-mir-375	*	< 0.0001	28638.7	1739.27	26899.5	6135.86	4.38397	39
hsa-mir-429		0.43293	178.86	211.756	-32.8956	41.5255	0.792179	40
hsa-mir-532		0.298194	798.969	969.966	-170.998	162.284	1.05369	41
hsa-mir-92a-2		0.0399419	5822.92	8259.04	-2436.12	1148.13	2.12182	41
hsa-mir-93	*	< 0.0001	3183.31	7875.17	-4691.87	863.43	5.43399	41
hsa-mir-99b		0.34135	30202.9	26313.6	3889.3	4040.07	0.962683	41

Supplementary Table 7. Multiple t-tests of miRNA expression in samples with/without tumor-specific insertions (found by Transpo-Seq), including all miRNAs expressed in lung cancer. Unpaired two-tailed t-tests, adjusting by FDR<0.01, were performed to calculate statistical significance.

	Discovery?	P value	Mean 0 insertions	Mean >1 insertions	Difference	SE of difference	t ratio	df
hsa-let-7a-1		0.98099	0.463243	0.46501	-0.0017664	0.0736816	0.0239734	41
hsa-let-7a-2		0.987214	0.462162	0.463356	-0.00119429	0.0740704	0.0161237	41
hsa-let-7a-3		0.958626	0.463335	0.467166	-0.00383084	0.0733935	0.0521959	41
hsa-let-7b		0.876557	0.469361	0.459521	0.00983987	0.0629417	0.156333	40
hsa-let-7c		0.0697986	0.351242	0.232458	0.118784	0.0637979	1.86188	41
hsa-let-7d		0.0759944	0.506158	0.395171	0.110987	0.0610043	1.81933	42
hsa-let-7e		0.7281	0.384512	0.409737	-0.0252252	0.0720638	0.35004	41
hsa-let-7f-2		0.780127	0.330059	0.310725	0.0193339	0.0687819	0.28109	39
hsa-let-7g		0.738525	0.585207	0.565964	0.0192428	0.057266	0.336025	42
hsa-let-7i		0.857094	0.478507	0.489858	-0.0113513	0.0626413	0.181212	41
hsa-mir-34a		0.576568	0.357295	0.399707	-0.0424121	0.0753306	0.563013	40
hsa-mir-34b		0.843279	0.150189	0.1639	-0.0137105	0.0689114	0.198959	41
hsa-mir-34c		0.722209	0.162988	0.190226	-0.0272382	0.0760927	0.357961	41
hsa-mir-200a		0.066558	0.304624	0.198382	0.106242	0.0563675	1.8848	41
hsa-mir-200b		0.406833	0.367692	0.313634	0.0540582	0.0644839	0.83832	40
hsa-mir-200c		0.937121	0.41371	0.40718	0.00652934	0.0822601	0.0793743	41
hsa-mir-429		0.525664	0.314785	0.273339	0.0414462	0.0647349	0.640245	40
hsa-mir-21		0.61918	0.638485	0.665902	-0.027417	0.054745	0.500814	41
hsa-mir-17		0.707371	0.390812	0.363903	0.0269092	0.071186	0.378012	41
hsa-mir-18a		0.211808	0.332274	0.236385	0.0958888	0.0755979	1.26841	41
hsa-mir-19a		0.0964777	0.220507	0.334859	-0.114352	0.0671789	1.70221	40
hsa-mir-19b-2		0.0194079	0.357518	0.522364	-0.164846	0.0676035	2.43842	39
hsa-mir-20a		0.740829	0.337519	0.359804	-0.0222848	0.0669096	0.333059	40
hsa-mir-92a-2		0.629718	0.424938	0.388063	0.036875	0.0759108	0.485767	41
hsa-mir-221		0.610987	0.309385	0.275763	0.0336223	0.0655927	0.512593	41
hsa-mir-222		0.835716	0.321192	0.335416	-0.0142234	0.0681416	0.208733	40

Supplementary Table 8. Multiple t-tests of lung cancer-related miRNA expression in samples after randomization of the number of insertions (found by Transpo-Seq). Unpaired two-tailed t-tests, adjusting by FDR<0.01, were performed to calculate statistical significance.

	Discovery?	P value	Mean 0 insertions	Mean >1 insertions	Difference	SE of difference	t ratio	df
hsa-let-7a-1		0.639935	0.543149	0.573305	-0.0301565	0.0638536	0.472276	32.0
hsa-let-7a-2		0.641723	0.539759	0.569778	-0.0300192	0.0639054	0.469744	32.0
hsa-let-7a-3		0.610677	0.545227	0.577777	-0.0325507	0.0633092	0.514155	32.0
hsa-let-7b		0.856758	0.473164	0.48882	-0.0156556	0.0860574	0.181921	33.0
hsa-let-7c		0.559296	0.338033	0.286321	0.0517125	0.0876397	0.590057	32.0
hsa-let-7d		0.611078	0.434326	0.393702	0.0406238	0.0791002	0.513574	32.0
hsa-let-7e		0.722865	0.558457	0.586251	-0.0277937	0.0776863	0.357769	32.0
hsa-let-7f-2		0.704482	0.550936	0.522865	0.0280716	0.0733327	0.382798	31.0
hsa-let-7g		0.16717	0.476528	0.35792	0.118608	0.0839122	1.41348	32.0
hsa-let-7i		0.804942	0.481651	0.50096	-0.0193089	0.0775417	0.249013	32.0
hsa-mir-34a		0.311754	0.515715	0.425365	0.0903507	0.0879076	1.02779	32.0
hsa-mir-34b		0.298895	0.223496	0.147123	0.0763732	0.0723623	1.05543	33.0
hsa-mir-34c		0.210877	0.28382	0.176961	0.106859	0.0837481	1.27596	33.0
hsa-mir-200a		0.629332	0.420852	0.46082	-0.0399678	0.0819848	0.487503	31.0
hsa-mir-200b		0.347076	0.354562	0.432957	-0.0783956	0.0821086	0.95478	31.0
hsa-mir-200c		0.216411	0.512961	0.405404	0.107558	0.0852929	1.26104	32.0
hsa-mir-429		0.132548	0.28543	0.433004	-0.147574	0.0955307	1.54478	31.0
hsa-mir-21		0.0212201	0.5502	0.369336	0.180864	0.0746397	2.42317	32.0
hsa-mir-17		0.337206	0.279988	0.187778	0.0922098	0.0946403	0.974319	32.0
hsa-mir-18a		0.770611	0.209792	0.181271	0.0285214	0.0969651	0.294141	31.0
hsa-mir-19a		0.500835	0.258057	0.200665	0.057392	0.084289	0.680896	32.0
hsa-mir-19b-2		0.757504	0.30268	0.271613	0.0310671	0.0997864	0.311336	33.0
hsa-mir-20a		0.589296	0.273441	0.221664	0.0517761	0.0949707	0.54518	33.0
hsa-mir-92a-2		0.15013	0.405995	0.277911	0.128084	0.0868001	1.47563	31.0
hsa-mir-221		0.10285	0.435371	0.29448	0.140891	0.0839777	1.67772	33.0
hsa-mir-222		0.341062	0.300849	0.227858	0.0729905	0.0755236	0.966459	32.0

Supplementary Table 9. Multiple t-tests of lung cancer-related miRNA expression in breast cancer samples with/without tumor-specific insertions (found by Transpo-Seq). Unpaired two-tailed t-tests, adjusting by FDR<0.01, were performed to calculate statistical significance.

Primer name	Sequence (5’ to 3’)
Let7aAAA	TGAGGTAGTAGGTTGTATAGTTAAA
Let7bAAA	TGAGGTAGTAGGTTGTGTGGTTAAA
miR34aAAA	TGGCAGTGTCTTAGCTGGTTGTAAA
Let7-ORF2PCRafw	CACAAGCATTCTTATACACC
Let7-ORF2PCRbrv	TATGGCTAGCCAGTTTTCCC
Let7-ORF2PCRa_PG2rv	AAAATCCCCCAACGTGATTCCTCCAGCTTTGTTC
Let7-ORF2PCRb_PG2fw	GAGGAATCACGTTGGGGGATTTTAAACTATACTAC
N51 Fw	GAATGATTTTGACGAGCTGAGAGAA
N51 Rv	GTCCTCCCGTAGCT CAGAGTAATT
SV40 Fw	TGGACAAACCACAACTAGAATGC
SV40 Rv	TTGCAGCTTATAATGGTTAC
HMGA2 Fw	TTGCTGCCTTTGGGTCTTCC
HMGA2 Rv	CAGCGCCTCAGAAGAGAGGACG
DICER Fw	AGTGGTAGGCTTTCACACAG
DICER Rv	AGAAAGGACCCATTGGTGAG
GAPDH Fw	TGCACCACCAACTGCTTAGC
GAPDH Rv	GGCATGGACTGTGGTCATGAG
NEOjunct2 Fw	TGCCTCGTCCTGAAGCTC
NEOjunct2 Rv	CAATCGGCTGCTCTGATG
CMV Fw	ACTGCCAAGTAGGAAAGTCCCA
CMV Rv	ATGCCAAGTACGCCCCCTAT
EBNA-1 Fw	CGTCATCTCCGTCATCACC
EBNA-1 Rv	AGATTTGCCTCCCTGGTTTC
genomicGAPDH Fw	CGTTTCCCAAAGTCCTCCTGT
genomicGAPDG Rv	AGGTGATCGGTGCTGGTTC
scrb Fw	TACAGTTGCGTTGTAGAACGATATAGAGGAACTACGCAGTAAGGTA
scrb Rv	TACCTTACTGCGTAGTTCCTCTATATCGTTCTACAACGCAACTGTA
bs2 Fw	TACGAACAAAGCTGGAGGCATCACACTACCTGACTTCAAACTAGTA
bs2 Rv	TACTAGTTTGAAGTCAGGTAGTGTGATGCCTCCAGCTTTGTTCGTA
8mer Fw	TACGAACAAAGCTGGAGGCATCACTCTACCTCACTTCAAACTAGTA
8mer Rv	TACTAGTTTGAAGTGAGGTAGAGTGATGCCTCCAGCTTTGTTCGTA
Let7-ORF2PCRa_8mer	GAAGTGAGGTAGAGTGATGCCTCCAGCTTTGTTC
Let7-ORF2PCRb_8mer	GAGGCATCACTCTACCTCACTTCAAACTATACTAC
Let7-Bcl1-ORF2bs-PCRaFw	TGGATTCACAGCCGAATTCTACC

Supplementary Table 10. List of primers used in this study.
