# Supplementary material for: The tumor suppressor microRNA let-7 inhibits human LINE-1 retrotransposition
Source: Nat Commun. 2020 Nov 11;11:5712. doi: 10.1038/s41467-020-19430-4 (PMC7658363; doi:10.1038/s41467-020-19430-4)
Supplement: Supplementary file 3 — Reporting Summary [file 41467_2020_19430_MOESM3_ESM.pdf]

nature research

Corresponding author(s): Sara R. Heras

Last updated by author(s): Sep 16, 2020

Reporting Summary

Nature Research wishes to improve the reproducibility of the work that we publish. This form provides structure for consistency and transparency in reporting. For further information on Nature Research policies, see Authors & Referees and the Editorial Policy Checklist .

Statistics

For all statistical analyses, confirm that the following items are present in the figure legend, table legend, main text, or Methods section.

n/a	Confirmed	
☐	☒	The exact sample size (*n*) for each experimental group/condition, given as a discrete number and unit of measurement
☐	☒	A statement on whether measurements were taken from distinct samples or whether the same sample was measured repeatedly
☐	☒	The statistical test(s) used AND whether they are one- or two-sided<br>*Only common tests should be described solely by name; describe more complex techniques in the Methods section.*
☒	☐	A description of all covariates tested
☐	☒	A description of any assumptions or corrections, such as tests of normality and adjustment for multiple comparisons
☐	☒	A full description of the statistical parameters including central tendency (e.g. means) or other basic estimates (e.g. regression coefficient) AND variation (e.g. standard deviation) or associated estimates of uncertainty (e.g. confidence intervals)
☐	☒	For null hypothesis testing, the test statistic (e.g. *F*, *t*, *r*) with confidence intervals, effect sizes, degrees of freedom and *P* value noted<br>*Give P values as exact values whenever suitable.*
☒	☐	For Bayesian analysis, information on the choice of priors and Markov chain Monte Carlo settings
☒	☐	For hierarchical and complex designs, identification of the appropriate level for tests and full reporting of outcomes
☐	☒	Estimates of effect sizes (e.g. Cohen's *d*, Pearson's *r*), indicating how they were calculated

Our web collection on statistics for biologists contains articles on many of the points above.

Software and code

Policy information about availability of computer code

Data collection	We used the Genomic Data Commons (GDC) Data Transfer Tool Client for downloading data from TGCA repository. It is a standard client-based mechanism in support of high-performance data downloads.
Data analysis	The analysis of non-reference Mobile Element Insertions (MEIs) in whole genome sequencing (WGS) data were performed using the software package MELT: The Mobile Element Locator Tool version 2.1.5.<br>The GraphPad Prism 6 software was used for statistical analysis.

For manuscripts utilizing custom algorithms or software that are central to the research but not yet described in published literature, software must be made available to editors/reviewers. We strongly encourage code deposition in a community repository (e.g. GitHub). See the Nature Research guidelines for submitting code & software for further information.

Data

Policy information about availability of data

All manuscripts must include a data availability statement. This statement should provide the following information, where applicable:

- Accession codes, unique identifiers, or web links for publicly available datasets
- A list of figures that have associated raw data
- A description of any restrictions on data availability

Data sets used in Figure 1 and Supplementary Figure 1 are detailed in Supplementary Table I. All data is available from the GDC legacy archive (https://portal.gdc.cancer.gov/legacy-archive). Though most data files can be accessed without requiring access approval, WGS files need a special request due to their potential identification information. Researchers interested in accessing to restricted data can obtain authorization following the instructions in https://gdc.cancer.gov/access-data/obtaining-access-controlled-data. The raw data underlying Figures 1-5 and Supplementary Figures 1-5 are provided as a Source Data file.

Field-specific reporting

Please select the one below that is the best fit for your research. If you are not sure, read the appropriate sections before making your selection.

- [x] Life sciences
- [ ] Behavioural & social sciences
- [ ] Ecological, evolutionary & environmental sciences

For a reference copy of the document with all sections, see nature.com/documents/nr-reporting-summary-flat.pdf

Life sciences study design

All studies must disclose on these points even when the disclosure is negative.

Sample size	To study the correlation between microRNA expression and the accumulation of tumor specific L1 insertions in Fig 1, all the lung adenocarcinoma (LUAD) and lung squamous cell carcinoma (LUSC) samples with paired tumor-normal solid tissue whole-genome sequencing (WGS) data and tumor miRNAs expression files available in TGCA were analysed.The sample sizes in the other experiments were determined based on our experience and general standards in the field. Most of the experiments were repeated three times (n=3). Similar methods can be found in the ref.45 , ref. 86. For the blotting analysis, RNA-inmunoprecipitation assay and endogenous microRNA expression quantification the representative results are from two or three independently performed experiments. A Similar method could bef ound in ref. 45. The number of independent experiments is indicated in each figure legend.
Data exclusions	Four samples in LUSC (TCGA-60-2695, TCGA-60-2722) and LUAD (TCGA-55-1594, TCGA-55-1596) were excluded from analysis for figure 1 and Supplementary Figure 1 because they did not pass our quality controls. These controls are based on MELT filtering and its effect on reference polymorphic insertion calls found both in tumour and normal tissue. For filtering we applied the highest MELT quality scores (ASSESS=5, FILTER=PASS; see Ref. 48); Supplemental Table S2 and Supplemental Methods) and excluded insertion calls under three split reads. Samples where common reference polymorphic calls number was abruptly reduced under a 10% after filtering were excluded. The rationale behind it lies in the fact that common reference polymorphic calls are real insertions found in both tumor and normal tissue and therefore they can be used as a measure of how filter parameters affect to putative de novo insertions. This 10% criteria was pre-established to exclude low quality samples.
Replication	Experiments are reliably reproduced. Generally, experiments were performed at least three times, unless otherwise noted in figure legends. The correlation between microRNA expression and the accumulation of tumor specific L1 insertions identified by MELT was replicated using the number of tumor specific L1 insertions obtained by Helman et al. in a group of different samples and using a different tool (Transpo-seq)
Randomization	Randomization is not relevant to our study because the study does not involve the allocation of samples into experimental groups
Blinding	No blinding was performed in this study because group allocation was not involved in our study. The researchers were not blinded during data collection because most of the measurements were performed using instruments, or were quantitative in nature (RT-qPCR,blots or numbers of colonies on a plate).

Reporting for specific materials, systems and methods

We require information from authors about some types of materials, experimental systems and methods used in many studies. Here, indicate whether each material, system or method listed is relevant to your study. If you are not sure if a list item applies to your research, read the appropriate section before selecting a response.

Materials & experimental systems

n/a	Involved in the study	
	x	Antibodies
	x	Eukaryotic cell lines
x		Palaeontology
x		Animals and other organisms
x		Human research participants
x		Clinical data

Methods

n/a	Involved in the study	
x		ChIP-seq
	x	Flow cytometry
x		MRI-based neuroimaging

Antibodies

Antibodies used	polyclonal rabbit anti L1-ORF1p (provided by Dr. Oliver Weichenrieder, Max-Planck, Germany), a policlonal rabbit anti L1-ORF1p SE-6798 (Gael Cristofari lab), anti-FLAG M2 mouse (Sigma, F3165), anti HMGA2 (ab97276, Abcam), anti DICER (#3363, Cell Signalling technology), anti-tubulin (D-10)(sc-5274 Santa Cruz), anti-actin (A2228, Sigma), rat monoclonal anti-GFP, 3H9 clone (3h9-20,Chromotek). For chemiluminescent detection we used anti rabbit HRP (Cell Signaling) or anti mouse HRP (Cell Signaling).
Validation	The polyclonal rabbit anti L1-ORF1p provided by Dr. Oliver Weichenrieder has been previously cited by Benitez-Guijarro M (2018) EMBO J.;37(15) and by Macia A, et al. (2017). Genome Res. 27(3):335-348.The rabbit polyclonal antibody against human ORF1p SE-6798 was validated for ectopic expression of ORF1p (Monot et al. PLoS Genet 2013) and then further validated by shRNA and

western-blot/RNA-seq for endogenous expression in Philippe et al. eLife 2016.The specificities of the anti-HMGA2, anti-DICER, anti-tubulin and anti-actin antibodies were verified as single intense bands of the expected molecular weight by western blotting (Supplementary Figure 6). The specificities of anti-flag and anti-GFP was
verified using flag-tagged protein and GFP protein, respectively, in western blotting and inmunoprecipitation (Supplementary Figure 6).The comercial antibodies can be used in multiple human cell lines according the manufacturer's website where relevant citations are provided.

Eukaryotic cell lines

Policy information about cell lines

Cell line source(s)	HEK293T, PA-1, HeLa and U2OS cells were originally obtained from ATCC and were provided by Drs Jose Luis Garcia-Perez (IGMM, Edinburgh, UK) and John V. Moran (University of Michigan, US). Lung cancer cell lines (A549, SK-MES-1) were provided by Dr Pedro Medina (GENYO, Spain). Flp-In-293 cells were generated previously in Ian Adam's lab (Maclennan et al, (2018) eLife).
Authentication	The identity of each cell line was confirmed by Short Tandem Repeat profiling.
Mycoplasma contamination	All cell lines tested negative for mycoplasma contamination.
Commonly misidentified lines (See ICLAC register)	No commonly misidentified cell lines were used in the study.

Flow Cytometry

Plots

Confirm that:

- [x] The axis labels state the marker and fluorochrome used (e.g. CD4-FITC).
- [x] The axis scales are clearly visible. Include numbers along axes only for bottom left plot of group (a 'group' is an analysis of identical markers).
- [x] All plots are contour plots with outliers or pseudocolor plots.
- [x] A numerical value for number of cells or percentage (with statistics) is provided.

Methodology

Sample preparation	Transfected HeLa and HEK293T cells were detached with TrypLE Express (Gibco) for 5-10 min at 37ºC, pelleted 4 min at 200g, resuspended in 1X PBS with 5% FBS and 5mM EDTA, and passed through a 70µM filter
Instrument	Cytometers used were FACS Canto (BD) for EGFP-based retrotransposition assays, and FACSAria (BD) for pVAN583-based experiments.
Software	Experiments were analyzed with FlowJo software (LLC) version 10
Cell population abundance	For each replicate, 10^5 cells were passed through the cytomerer. Only live and transfected cells (7AAD- and GFP+, between 3600 and 9300 cells) were used for %mCherry analysis, which was performed using FlowJo software (LLC). Controls were used to set the threshold for each fluorescent channel of detection: untransfected cells, and cells expressing either GFP only or mCherry only.
Gating strategy	Single Cells were selected using SSC and FSC gates, GFP+ cells were selected using FITC-A and SSC gates and mCherry+ cells were selected using PE-Texas RedA and SSC gates.

- [x] Tick this box to confirm that a figure exemplifying the gating strategy is provided in the Supplementary Information.
