# Supplementary material for: The tumor suppressor microRNA let-7 inhibits human LINE-1 retrotransposition
Source: Nat Commun. 2020 Nov 11;11:5712. doi: 10.1038/s41467-020-19430-4 (PMC7658363; doi:10.1038/s41467-020-19430-4)
Supplement: Supplementary file 4 — Description of Additional Supplementary Files [file 41467_2020_19430_MOESM4_ESM.pdf]

Description of Additional Supplementary Files

File Name: Supplementary Data 1
Description: Description of tumor-specific L1 insertions found by MELT.’
